# HPV E2, E4, E5 drive alternative carcinogenic pathways in HPV positive cancers

**DOI:** 10.1038/s41388-020-01431-8

**Published:** 2020-08-26

**Authors:** Shuling Ren, Daria A. Gaykalova, Theresa Guo, Alexander V. Favorov, Elana J. Fertig, Pablo Tamayo, Juan Luis Callejas-Valera, Mike Allevato, Mara Gilardi, Jessica Santos, Takahito Fukusumi, Akihiro Sakai, Mizuo Ando, Sayed Sadat, Chao Liu, Guorong Xu, Kathleen M. Fisch, Zhiyong Wang, Alfredo A. Molinolo, J. Silvio Gutkind, Trey Ideker, Wayne M. Koch, Joseph A. Califano

**Affiliations:** 1 Moores Cancer Center, University of California San Diego, La Jolla, CA USA; 2 Department of Otolaryngology—Head and Neck Surgery, Beijing Friendship Hospital, Capital Medical University, Beijing, China; 3 Department of Otolaryngology—Head and Neck Surgery, Johns Hopkins Medical Institutions, Baltimore, MD USA; 4 Division of Oncology Biostatistics, Department of Oncology, Johns Hopkins Medical Institutions, Baltimore, MD USA; 5 Laboratory of Systems Biology and Computational Genetics, Vavilov Institute of General Genetics, Russian Academy of Sciences, Moscow, Russia; 6 Department of Medicine, University of California San Diego, La Jolla, CA USA; 7 Cancer Biology and Immunotherapies group, Sanford Research, Sioux Falls, SD USA; 8 Division of Otolaryngology—Head and Neck Surgery, Department of Surgery, University of California San Diego, La Jolla, CA USA

**Keywords:** Cancer models, Head and neck cancer, Tumour virus infections, Sequencing

## Abstract

The dominant paradigm for HPV carcinogenesis includes integration into the host genome followed by expression of E6 and E7 (E6/E7). We explored an alternative carcinogenic pathway characterized by episomal E2, E4, and E5 (E2/E4/E5) expression. Half of HPV positive cervical and pharyngeal cancers comprised a subtype with increase in expression of E2/E4/E5, as well as association with lack of integration into the host genome. Models of the E2/E4/E5 carcinogenesis show p53 dependent enhanced proliferation in vitro, as well as increased susceptibility to induction of cancer in vivo. Whole genomic expression analysis of the E2/E4/E5 pharyngeal cancer subtype is defined by activation of the fibroblast growth factor receptor (FGFR) pathway and this subtype is susceptible to combination FGFR and mTOR inhibition, with implications for targeted therapy.

## Introduction

Human papillomavirus (HPV) is a ~7.9 kb, non-enveloped, double-stranded, circular DNA virus that has a specific tropism for squamous epithelium, and persistent infection with oncogenic HPV subtypes is associated with development of cancers of the cervix, oropharynx, vagina, vulva, penis, and nasal cavity. HPV is responsible for 4.5% of all cancers worldwide (630,000 new cancer cases per year) [1]. High-risk HPV16 accounts for the largest proportion of HPV positive tumors, with HPV16 associated with 85% of HPV positive head and neck squamous cell carcinoma (HNSCC) and HPV 16 and 18 in 70% of cervical cancers [1, 2]. HPV-driven HNSCC display distinct biological and clinical features, including a favorable clinical prognosis [3]. HPV positive cancers have less somatic alterations and protein expression change comparing with HPV negative cancers [4]. Interestingly, both cervical squamous cell carcinoma (CESC) and HPV positive HNSCC show an increase rate of *PIK3CA* mutations in comparison to other solid tumors, and the PIK3CA-AKT-mTOR pathway is the most frequent dysregulated signaling pathway in HNSCC, including >80% of all HPV negative and HPV positive cases [4, 5].

After infection, HPV viral DNA may remain in a latent infective state in affected basal epithelial cells that can transit to a productive neoplastic infection, followed by transformation over many years to invasive cancer. A critical step in progression to cancer is thought to be the integration of HPV DNA sequence into the host genome [6], associated with copy number alterations (CNA), mRNA transcript abundance and splicing, and both inter- and intrachromosomal rearrangements [7]. This is associated with expression of the E6 and E7 (E6/E7) oncoproteins, which would respectively inactivate p53 and members of the pRb family, interfering with the cellular control mechanisms of the cell cycle [8] as well as other cellular networks including the PIK3CA-AKT-mTOR pathway [9]. However, recent data indicate that integration can be in a nonuniform manner with variation in the size and region of HPV genome integration as well as host genome integration site [6]. In addition, the functional relevance of other HPV gene products is relatively understudied. HPV E2 plays a key role in viral genome replication, RNA transcription, as well as partitioning viral epigenomes during replication [10, 11]. HPV E4 has been shown to induce G2/M arrest and facilitate E6/E7 viral amplification [12], but also play a role in viral genome amplification and maintenance of MAPK activation and may interact and stabilize E2 [13, 14]. HPV16 E5 has been shown to cooperate with E7 in cell transformation, inhibit immune response, increase cell motility, but has also been shown to inhibit proliferation [15, 16]. As a part of a TCGA team led by Parfenov et al. [6], we found a substantial subset of HPV positive HNSCC which demonstrated minimal expression of HPV16 E6/E7, but dramatic increase in expression of E2, E4, and E5 (E2/E4/E5) genes, as well as exclusive association of E2/E4/E5 expression with lack of HPV16 integration into the host genome. HPV positive HNSCC with or without integrated HPV also displayed different patterns of DNA methylation and both human and viral gene expression [6]. These data imply that there may be an alternative mechanism of HPV viral oncogenesis that does not depend on E6/E7 expression or viral integration, but may be driven by episomal E2/E4/E5 expression.

In this study, we validated the oncogenic potential of a non-integrated, E2/E4/E5 expressing subtype in HPV positive HNSCC as well as in cervical carcinoma. To provide functional insights into the HPV16 E2/E4/E5 subtype, we mimicked HPV16 E2/E4/E5 carcinogenesis using in vitro and in vivo models, and performed single sample gene set enrichment analysis (ssGSEA) to map key downstream networks and identify novel therapeutic targets for HPV positive HNSCC. This approach defines a novel mechanism of HPV carcinogenesis driven by non-integrated, E2/E4/E5 expression, and will provide opportunities for novel, effective therapies for these tumors.

## Results

### Discovery of HPV gene expression subtypes

**Fig. 1:**
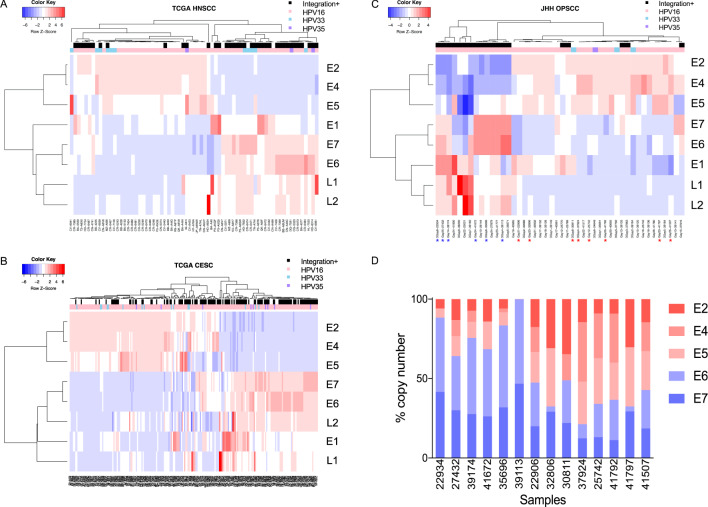
Discovery of HPV gene expression subtypes. **a**–**c** Heatmaps of HPV gene expression (genes in rows and samples in columns). The expression levels of each gene in each sample was indicated gradually from blue to red. Samples were annotated in integration status and HPV types. According to the expression of E2/E4/E5 and E6/E7, there are two major clusters: most of samples in E2/E4/E5 high expression group were non-integrated, while samples in E6/E7 high expression group were integrated. **a** TCGA HPV positive HNSCC dataset. **b** TCGA CESC dataset. **c** JHH HPV positive OPSCC dataset. Fourteen samples were chosen for RT-qPCR validation. Corresponding samples were signed with asterisks (E2/E4/E5 group in red and E6/E7 group in blue). **d** RT-qPCR validation of RNAseq results. HPV gene expression were validated with RT-qPCR using absolute quantitation method. The RT-qPCR result is completely concordant with RNAseq result.

In order to answer the question of whether significant HPV gene expression subtypes exist in HPV positive cancer, we explored the expression patterns of HPV genes and correlated them with the presence or absence of HPV integration in the host genome. We aligned RNAseq data of HPV positive HNSCC in the most recent updated TCGA HNSCC dataset to high-risk HPV16 (*n* = 55, 79.71%), HPV33 (*n* = 11, 15.94%), and HPV35 (*n* = 3, 4.35%) genomes independently, and calculated read counts of each gene (Supplementary Table S1). We performed unsupervised clustering depending on sum of counts of each HPV gene, and annotated with integration status and HPV types. We found two obvious clusters: tumors with integrated HPV were characterized by high expression of E6/E7 and low expression of E2/E4/E5, while nonintegrated tumors had high expression of E2/E4/E5 and low expression of E6/E7 (Fig. 1a). This association between HPV gene expression and integration status was validated in the TCGA CESC dataset (Fig. 1b) and an independent Johns Hopkins Hospital (JHH) HPV positive oropharynx squamous cell carcinoma (OPSCC) dataset (Fig. 1c), which were concordant with our earlier findings [6]. We further validated the RNAseq data by choosing 14 primary tumor samples from JHH dataset and employing quantitative real-time polymerase chain reaction (RT-qPCR) using an absolute quantitation method (Fig. 1d) demonstrating complete concordance between RNAseq and RT-qPCR data in six samples with high expression in E6/E7, and eight samples with high expression of E2/E4/E5. Sequences of each primers and probe set are provided in Supplementary Table S2.

We employed unsupervised clustering of HPV positive HNSCC samples in TCGA dataset (Supplementary Fig. S1a) and HPV positive OPSCC samples in JHH dataset (Supplementary Fig. S1b), respectively, using all statistically significant outlier genes in E2/E4/E5 activated, nonintegrated samples and E6/E7 activated, integrated samples compared with normal samples. Annotations were made with HPV integration status and gene expression. The E2/E4/E5 positive dominated clade tend to have higher expression across these genes than the integration positive dominated clade. Of note, unsupervised clustering of somatic gene expression was more strongly associated with E2/E4/E5 and E6/E7 expression status, rather than integration, implying that clusters of HPV gene expression strongly drive whole genomic expression. Using mRNA expression of significant outlier genes in E2/E4/E5 activated, nonintegrated samples and E6/E7 activated, integrated samples, we found subsets of genes with significantly distinct patterns of mRNA expression (Supplementary Fig. S2) between E2/E4/E5 and E6/E7 subtypes in the TCGA HNSCC dataset. To study the influence of HPV gene expression on the host cell, we correlated the patterns of HPV gene expression with disease-free survivals of HNSCC patients, and compared the patterns of alteration events of HNSCC and CESC in two different HPV gene expression subtypes. We chose significantly somatically mutated genes and explored the percentages of alteration events in each subtype using cBioPortal (http://www.cbioportal.org) in TCGA HNSCC dataset (Supplementary Fig. S3a) and CESC dataset (Supplementary Fig. S3b). Major mutations included *TP53*, *ZNF750*, *PIK3CA*, *EP300*, *PTEN*, *AGTR1*, *B2M*, *MUC4*, and *C3ORF70*, however, there were no differences in mutations and CNA between two subtypes. Furthermore, we investigated the expression of HPV genes subtype with tumor recurrence in TCGA HPV positive HNSCC cohort. The E2/E4/E5 group had a poorer prognosis compared with E6/E7 group (median survival: 60.94 months vs. 71.22 months), but this was not significant (*P* = 0.41) (Supplementary Fig. S4).

### Concurrent E2/E4/E5 expression contributes to cell proliferation in vitro

**Fig. 2:**
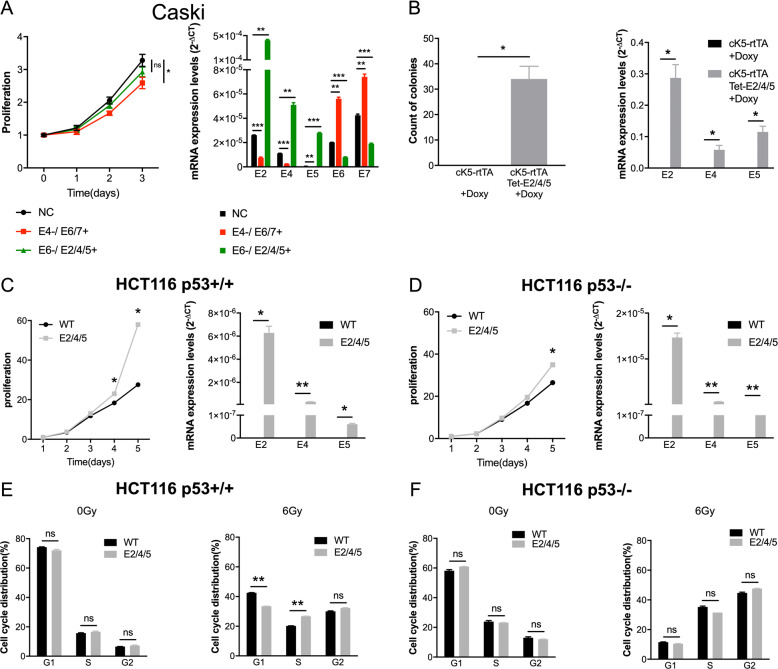
HPV16 E2/E4/E5 attribute to cell proliferation and attribute to cell cycle in HCT116 p53+/+ cells in vitro. **a** Growth curve on the left and RT-qPCR validation on the right. Co-transfection of E4 siRNA and E6/E7 plasmid (E4−/ E6/7+) shows growth inhibition in HPV positive cervical cancer cell line Caski compared with negative controls (NC) (*P* < 0.05). **b** Primary epithelia of cK5-rtTA/Tet-E2/E4/E5 mice showed colony formation progress. Primary tongue epithelia were collected from cK5-rtTA and cK5-rtTA/Tet-E2/E4/E5 transgenic mice models and were cultured with 33 ng/ml doxycycline for 2 weeks. E2/E4/E5 are highly expressed in the oral keratinocytes compared with control cells both treated with doxycycline. **c**–**f** HCT116 p53+/+ E2/E4/E5 stable expression cell line and HCT116 p53−/− E2/E4/E5 stable expression cell line were established. **c**, **d** On the left, proliferation in HCT116 p53+/+ cells and HCT116 p53−/− cells were analyzed. On the right, stable expression of E2/E4/E5 were validated using RT-qPCR. **e**, **f** Cell cycle was detected by FACS with PI staining. The percentages of different cell cycle phases in HCT116 p53+/+ cells and HCT116 p53−/− cells were analyzed using FlowJo. Untreated and irradiated HCT116 cells (6 Gy) were collected 24 h after treatment. Data represent mean ± SEM for three independent experiments with each experiment having five replicates. *P* values were calculated using two-sided Student *t* test. ns no significance; *P < 0.05; **P < 0.01; ***P < 0.001.

HPV gene transcripts include complex alternative splicing arrangements with inclusion of multiple genes in a single transcript [17, 18]. When attempting knockdown of single HPV genes (E2, E4, E6, or E7) in cell line systems, we noted global knockdown of all HPV genes rendering conventional siRNA strategies impossible. To investigate the effect of differential knockdown of E2/E4/E5 vs. E6/E7 transcripts, we used HPV positive CESC cell line Caski, which expresses all HPV16 genes and contains both episomal and integrated HPV16 genome [19]. To force either E6/E7 or E2/E4/E5 knockdown, we co-transfected with E4 siRNA in a pCEFL2 E6/E7 background or E6 siRNA in a pCEFL2 E2/E4/E5 background. RT-qPCR was subsequently performed to validate the transfection effect (Fig. 2a, right). Inhibition of E4 suppressed the growth of Caski cells regardless of the upregulation of E6/E7 compared to controls (*P* < 0.05; Fig. 2a, left). This demonstrated that E2/E4/E5 knockdown was more effective than E6/E7 knockdown in suppressing growth in Caski cells. We have done cell line work in additional HNSCC cell lines. We upregulated E2/E4/E5 expression in two types of HPV negative HNSCC cell lines, and we downregulated E4 expression in two types of HPV positive HNSCC cell lines (Supplementary Fig. S5). However, there is no significant change of growth after expressing E2/E4/E5 in HPV negative HNSCC cell lines. And in one out of two HPV positive HNSCC cell lines, we can find the cell growth was significantly decreased after inhibiting E2/E4/E5 expression (*P* < 0.05).

To define the effects of E2/E4/E5 further, we conditionally expressed the E2/E4/E5 genes using a doxycycline inducible cytokeratin 5 promoter—reverse tetracycline transactivator (cK5-rtTA) system as described [20]. We isolated primary normal oral keratinocytes from tongue epithelia from cK5-rtTA mice and cK5-rtTA/Tet-E2/E4/E5 mice. E2/E4/E5 expression significantly enhanced the colony number and colony size of primary oral keratinocytes compared with control cells upon Tet activation using doxycycline (*P* < 0.05; Fig. 2b).

### E2/E4/E5 induce proliferation and reduce cell cycle G1 arrest after irradiation in a p53-dependent manner

A hallmark of HPV mediated carcinogenesis is inhibition of p53 and Rb through the actions of E6 and E7, respectively. The definition of a clinical HPV cancer phenotype that preferentially works through E2/E4/E5 expression implies that these genes may also affect key cell cycle components and DNA damage response mediators like p53, and prior data indicate that HPV16 E2 binds p53 [21, 22]. To explore the hypothesis that E2/E4/E5 expression may also affect p53 function, we chose a well characterized cell line model of p53 function. The HCT116 p53+/+ and p53−/− cell lines were transfected with E2/E4/E5 overexpressed lentivirus and selected with puromycin. Both p53+/+ and p53−/− transfected with E2/E4/E5 showed growth induction compared with control cells (both *P* < 0.05; Fig. 2c, left; Fig. 2d, left). However, growth was more marked in HCT116 p53+/+ compared to p53−/− cells, indicating that E2/E4/E5 may enhance cell viability in part via a p53-dependent manner.

FACS was used to analyze the cell cycle distribution and apoptosis of irradiated and untreated cells. Experimental cells were irradiated with a dose of 6 Gy ^137^Cs and collected after 24 h at the same time with control cells to monitor cell cycle changes (Fig. 2e, f). Apoptotic cells were assayed at 48 h after irradiation (Supplementary Fig. S6). A rapid increase of irradiated p53+/+ and p53−/− cells in G2 was observed indicating a pronounced G2 arrest after irradiation. At the same time, the percentage of apoptotic cells increased dramatically in p53+/+ and p53−/− cells after irradiation. The percentage of cells in the G1 phase decreased dramatically following irradiation in E2/E4/E5 expressing p53+/+ cells compared with wild type p53+/+ cells (*P* < 0.01), and cells in the S-phase increased significantly (*P* < 0.01), which indicated that a G1-arrest was partially blocked by E2/E4/E5 (Fig. 2e). However, no significant change in E2/E4/E5 expressing HCT116 p53−/− cells could be seen after irradiation compared with wild type cells (Fig. 2f). At the same time, E2/E4/E5 reduced apoptosis slightly in response to radiation in p53+/+ cells (Supplementary Fig. S6).

Taken together, these data indicate that HPV16 E2/E4/E5 may induce proliferation and cell cycle progression in part through p53-dependent mechanisms, and impair activation of the DNA damage induced G1-S arrest in a p53-dependent manner.

### Expression of HPV16 E2/E4/E5 accelerates tumorigenesis in vivo

**Fig. 3:**
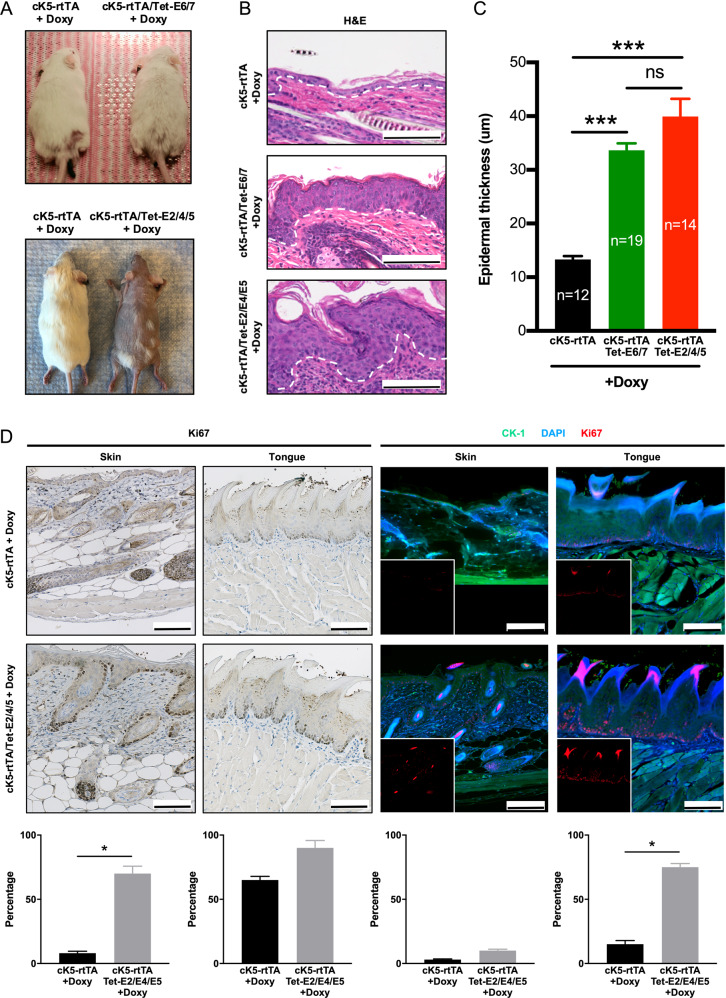
HPV16 E2/E4/E5 induced a marked skin hyperplasia in transgenic mice models. **a** Representative pictures of cK5-rtTA, cK5-rtTA/Tet-E2/E4/E5 and cK5-rtTA/Tet-E6/E7 mice 3 months after doxycycline induction. **b** Sections of back skin of cK5-rtTA, cK5-rtTA/Tet-E2/E4/E5, and cK5-rtTA/Tet-E6/E7 mice were fixed after doxycycline induction and stained with H&E. Scale bars: 100 μm. **c** Quantification of the thickness of epidermis was shown. Data represent mean ± SEM. *P* values were calculated using two-sided Student *t* test. ns no significance; ***P < 0.001. **d** Ki67 IHC and IF expression in skin and tongue of cK5-rtTA and cK5-rtTA/Tet-E2/E4/E5 mice. Fragment of cK5-rtTA skin showed few cells proliferating on thin epithelial lining and connective tissue. cK5-rtTA/Tet-E2/E4/E5 skin had higher Ki67 index (brown in IHC, red in IF). Tongues of cK5-rtTA and cK5-rtTA/Tet-E2/E4/E5 mice showed proliferating cells on basal layer of the parakeratinized stratified epithelial lining, with more pronounced Ki67 expression (brown in IHC, red in IF) on cK5-rtTA/Tet-E2/E4/E5 tongue. DAPI (blue) and cytokeratin 1 (green) were applied to contrast the tissues in IF. Insets showed single-channel for Ki67 in IF. Scale bars: 100 μm. Quantification of Ki-67 positive cells and related statistical differences were shown in the bottom. Data represent mean ± SEM. *P* values were calculated using two-sided Student *t* test. *P < 0.05.

We recently reported an inducible mouse model expressing high-risk HPV16 E6/E7 oncoproteins, targeted to the basal squamous epithelia in transgenic mice using a doxycycline inducible cK5-rtTA system [20]. After doxycycline induction, a rapid epidermal hyperplasia is observed with a remarkable expansion of the proliferative cells in the suprabasal layers. To explore the effects of HPV16 E2/E4/E5 expression on epithelial development and proliferation in vivo, we conditionally expressed E2/E4/E5 genes using an analogous doxycycline inducible cK5-rtTA system. Interestingly, we observed a similar rapid and clear induction of hair loss with an increase in skin thickness upon induction of HPV16 E2/E4/E5 expression (*P* < 0.001; Fig. 3a–c). This increase in thickness was identical to that found in cK5-rtTA/Tet-E6/E7 mice. Ki67 staining was used to identify proliferating cells on skin and tongue by IHC and immunofluorescence (IF) (Fig. 3d). For all anatomic cutaneous sites examined, cK5-rtTA/Tet-E2/E4/E5 mice demonstrated increased epithelial proliferation. cK5-rtTA/Tet-E2/E4/E5 skin epithelia had higher Ki67 index than cK5-rtTA epithelia (70% vs. 8% in IHC, 10% vs. 3% in IF), while cK5-rtTA/Tet-E2/E4/E5 tongue epithelia had higher Ki67 index than cK5-rtTA epithelia (90% vs. 65% in IHC, 75% vs. 15% in IF).

**Fig. 4:**
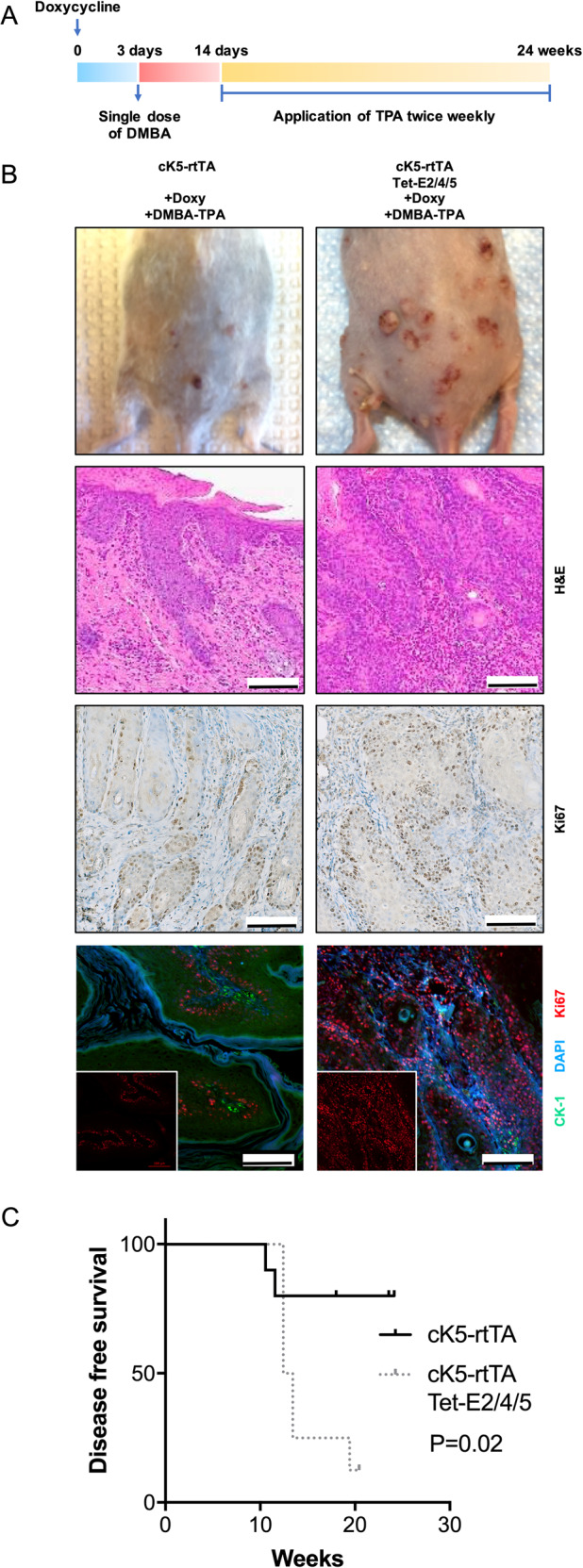
HPV16 E2/E4/E5 accelerates skin tumorigenesis in a two-stage carcinogenesis model. **a** Schematic representation of DMBA-TPA chemical induced carcinogenesis in cK5-rtTA mice and cK5-rtTA/Tet-E2/E4/E5 mice models. **b** All 10 cK5-rtTA mice did not exhibit any tumors, while 1 in 8 cK5-rtTA/Tet-E2/E4/E5 mice developed SCC at 14 weeks. Representative photos, H&E staining, Ki67 IHC, Ki67 IF pictures were shown. Lesions of cK5-rtTA and cK5-rtTA/Tet-E2/E4/E5 mice showed more pronounced Ki67 expression (brown in IHC, red in IF) on cK5-rtTA/Tet-E2/E4/E5. DAPI (blue) and cytokeratin 1 (green) were applied to contrast the tissues in IF. Insets showed single-channel for Ki67 in IF. Scale bars: 100 μm. **c** cK5-rtTA/Tet-E2/E4/E5 group has significant poorer prognosis compared with control group comparing the disease-free survivals (*P* = 0.02). *P* value was calculated using the Log-rank test.

To further explore the ability of E2/E4/E5 to drive tumor development, we used a classical two-stage carcinogenesis protocol [20]. cK5-rtTA mice and cK5-rtTA/Tet-E2/E4/E5 mice were treated with both 7,12-dimethylbenz(a)anthracene (DMBA), a skin carcinogen which causes multiple mutations and initiates tumorigenesis in the skin, and 12-O-tetradecanoylphorbol-13-acetate (TPA), a tumor promoter [23] (Fig. 4a). Lesions of cK5-rtTA/Tet-E2/E4/E5 mice showed more pronounced Ki67 expression compared to cK5-rtTA mice (65% vs. 50% in IHC, 90% vs. 35% in IF, Fig. 4b). We noted that cK5-rtTA/Tet-E2/E4/E5 mice developed tumors faster than the control group (*P* = 0.02) (Fig. 4c). These studies demonstrated that E2/E4/E5 expression accelerated carcinogenesis with exposure to carcinogen with early tumor development. Although a larger proportion of cK5-rtTA/Tet-E2/E4/E5 mice developed papillomas than cK5-rtTA mice, and occasionally developed SCC, this difference was not significant (Supplementary Fig. S7).

### HPV16 E2/E4/E5 expression is associated with a fibroblast growth factor receptor (FGFR) network activation signature

**Fig. 5:**
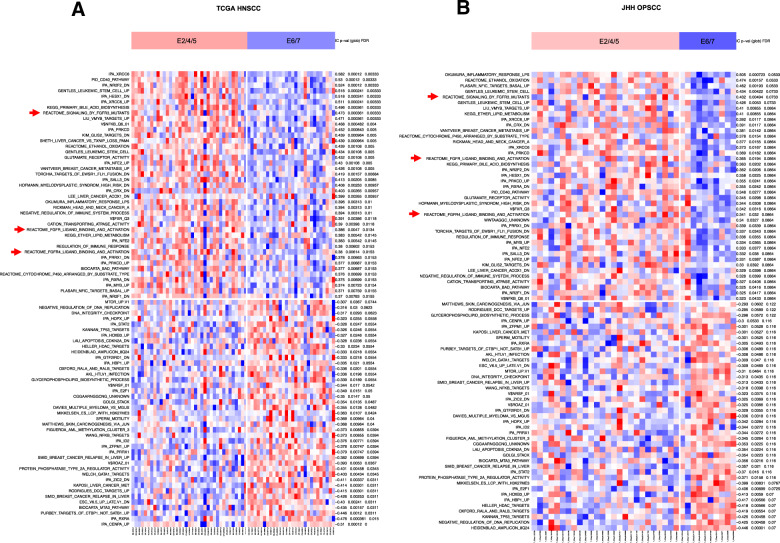
Expression of E2/E4/E5 is associated with differential activation of FGFR networks compared to E6/E7 expressing tumors. Heatmaps of significant gene sets correlated with HPV gene subtypes depending on ssGSEA of TCGA HPV positive HNSCC data (**a**) and JHH HPV positive OPSCC data (**b**) were shown. Forty-two overlapped most differentially enriched gene sets were chosen in E2/E4/E5 subtype, while 41 overlapped most differentially enriched gene sets were chosen in E6/E7 subtype. Despite significant overlap, we noted significant enrichment for three FGFR network gene sets (labeled with red arrows) in a subset of tumors with E2/E4/E5 expression that were validated in a separate JHH HPV positive OPSCC dataset.

To identify gene sets that correlated with each HPV gene expression subtype, we analyzed RNAseq RSEM in TCGA HPV positive HNSCC dataset and our JHH HPV positive OPSCC dataset using ssGSEA [24] and identified the gene sets most differentially enriched in each subtype. As we compared E2/E4/E5 subtype with E6/E7 subtype, we found 42 overlapped gene sets from top 500 gene sets enriched in E2/E4/E5 subtype and 41 gene sets from top 500 gene sets enriched in E6/E7 subtype (Supplementary Table S3). Heatmaps of overlapped significant gene sets expression in each dataset was shown (TCGA HNSCC: Fig. 5a; JHH OPSCC: Fig. 5b). Three significant gene sets defined by FGFR pathways activation were found enriched in E2/E4/E5 subtype, which included REACTOME signaling by FGFR3 mutants, REACTOME FGFR ligand binding and activation, and REACTOME FGFR4 ligand binding and activation. Selected gene sets such as mTOR up gene set, TP53 targets gene set, and apoptosis CDKN2A down gene set were enriched in E6/E7 subtype.

### HPV positive tumor growth associated with E2/E4/E5 expression is inhibited by FGFR inhibitor AZD4547 alone and in combination with mTOR inhibitor rapamycin

**Fig. 6:**
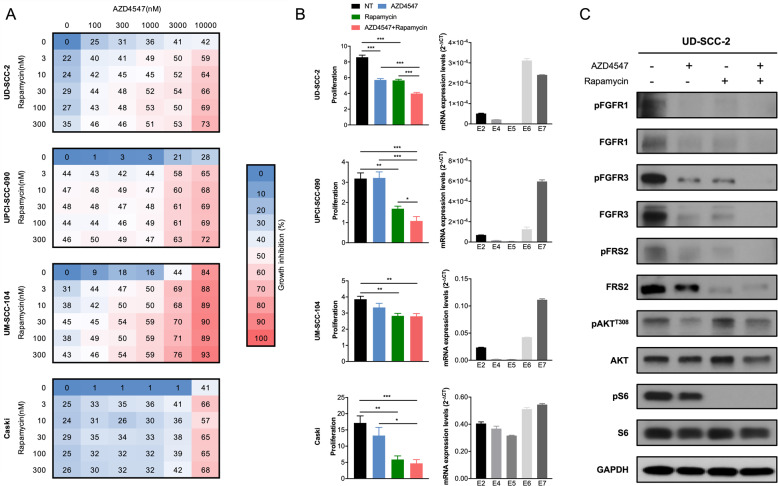
Tumor growth associated with HPV16 E2/E4/E5 expression is inhibited by FGFR inhibitor AZD4547 alone and in combination with mTOR inhibitor rapamycin. **a** AZD4547 synergizes with rapamycin in inhibiting cell viability of four HPV positive cancer cells. Four HPV positive cancer cell lines were treated with different concentration of AZD4547 (from 0 to 10,000 nM) and rapamycin (from 0 to 300 nM) at the same time. After 3 days, the viability was measured and growth inhibition was calculated normalized by the untreated cells (0 nM AZD4547, 0 nM rapamycin). **b** Growth inhibition histogram on the left and RT-qPCR on the right. Growth inhibition was measured at 3 days after treated with AZD4547 (1 uM), or/and rapamycin (200 nM), and normalized by the first day. Right histogram shows the expression of HPV16 E2, E4, E5, E6 and E7 in four HPV positive cancer cell lines. Data represent mean ± SEM. *P* values were calculated using two-sided Student *t* test. *P < 0.05; **P < 0.01; ***P < 0.001. **c** Western blot results of UD-SCC-2 cells after treated with AZD4547 or/and rapamycin for 3 days. AZD4547 induced decrease in pFGFR1, FGFR1, pFGFR3, FGFR3, pFRS2 and pAKT^T308^, and synergized with rapamycin in decrease in pFGFR1, FGFR1, pFGFR3, FGFR3, pFRS2, FRS2, pAKT^T308^ and pS6.

To determine the potential responsiveness to inhibition based on target networks identified by ssGSEA, we employed cell viability assays combining a FGFR inhibitor and a mTOR inhibitor in four HPV positive tumor cell lines with relatively high expression of E2/E4/E5 validated using RT-qPCR (Fig. 6a, b). AZD4547, a pan FGFR inhibitor, had growth inhibition effect on HPV positive cells, and synergized with rapamycin, a mTOR inhibitor, in inhibiting cell proliferation (Fig. 6a). We can find significant synergistic effects in all four cell lines (Chou Talalay combination index score < 0.5, Supplementary Fig. S8). When treated with AZD4547 or/and rapamycin, we noted significant inhibition of growth with either agent, and dramatic inhibition when used in combination (Fig. 6b). AZD4547 induced decrease in pFGFR1, FGFR1, pFGFR3, FGFR3, pFRS2 and pAKT^T308^, and synergized with rapamycin in decrease in pFGFR1, FGFR1, pFGFR3, FGFR3, pFRS2, FRS2, pAKT^T308^ and pS6 (Fig. 6c). While we were able to find synergistic effect in all cell lines, a high sensitivity to rapamycin in Caski and UM-SCC104 cell lines drove reduction in proliferation at higher therapeutic doses. Of note, response to AZD4547 was also noted in some cell lines with dominant E6/E7 expression (Supplementary Fig. S9), such that susceptibility to FGFR inhibition is not solely determined by HPV gene expression pattern in cell lines. Nevertheless, this demonstrates that computational discovery of network targets shows promise for identifying HPV E2/E4/E5 associated therapeutic susceptibility and synergies.

## Discussion

Many of the hallmark differences identified by existing studies relate to direct action of the viral oncogenes E6 and E7, which have been understood to be the dominant driving force in HPV carcinogenesis [4, 25]. However, we define an alternative HPV gene expression subtype in HPV positive tumors—E2/E4/E5 subtype, that is as common as E6/E7 expressing cancers in both HPV related cervix and head and neck cancer. We show that dominant E2/E4/E5 expression produces similar phenotypic biologic effects in vitro and in vivo, and therefore it is a useful and informative classification in HPV positive tumors that complements existing subtypes based on gene expression [26, 27] and integration [6, 28]. Interestingly, these tumors demonstrate p16 IHC staining, further indicating that they provide similar biologic outcomes and pathway alterations. However, we show that the E2/E4/E5 subtype is characterized by unique network alterations in oropharyngeal cancers that may be specifically targeted using existing therapeutic agents.

The etiological role of infection with high-risk HPVs in the vast majority of cervical carcinomas and a substantial proportion of head and neck cancers is well-established. HPV16 species group (alpha-9) of the alphapapillomavirus genus primarily contains HPV16, HPV33, HPV35. These HPVs account for more than 60% of cervical cancers [29, 30] and majority of HPV related head and neck cancers [31] worldwide. Reanalysis of the RNAseq data of HPV positive HNSCC and CESC in TCGA as well as a separate validation HPV positive OPSCC cohort, clearly define distinct clusters—an E2/E4/E5 expressing nonintegrated cluster and an E6/E7 expressing integrated cluster. Intuitively, this correlation exists since, during carcinogenesis parts of HPV DNA (E6/E7) integrate into the host genome [32]. Mixed expression patterns may suggest the presence of both integrated and episomal forms of HPV in the same sample [6]. Employing unsupervised clustering of HPV positive HNSCC samples in the TCGA dataset based on somatic gene expression, tumors are organized by E2/E4/E5 and E6/E7 expression status, rather than integration, implying that clusters of HPV gene expression, rather than integration, strongly drive whole genomic expression. Supervised clustering of mRNA expression levels show that difference between these two HPV gene expression subtypes does exist. However, investigation into mutation, CNA, and prognosis of these two subtypes did not show any significant difference, implying that these global genomic expression differences are more likely driven by HPV gene expression, rather than associated genome mutation, deletion, or amplification.

In order to detect the effect of only downregulating expression of E2/E4/E5 or E6/E7 respectively, we upregulated E6/E7 or E2/E4/E5 at the same time. E6/E7 are oncoproteins and have been attributed to tumorigenesis for decades [33–35]. The E6 oncoprotein binds to E6-associated protein (E6-AP), which interacts with p53, resulting in the rapid ubiquitin-dependent degradation of p53 [36]. E7 interacts with Rb family members, which control the G1–S phase transition by regulating the activity of the E2F family of transcription factors [37, 38]. For E2/E4/E5, conflicting data exist [21, 39–41], however, our study shows that concurrent E2/E4/E5 expression is key in the development of HPV positive cancers in vitro. By further exploring this mechanism, we found that the effects of E2/E4/E5 on cell growth and cell cycle are p53-dependent, consistent with findings by Massimi et al. [22] and Tan et al. [42]. We created a HPV16 E2/E4/E5 inducible mouse model under the control of cK5 promoter that rapidly develops skin hyperplasia, which includes the increase of proliferative cells in the basal epithelial layer with a profound expansion of the proliferative cells in the suprabasal layers. At the same time, E2/E4/E5 expression causes a dramatic increase in the clonogenic capacity in primary oral keratinocytes from cK5-rtTA mice model, thus supporting of E2/E4/E5 expression as contributing to epithelial cell proliferation. Using a well-established DMBA-TPA two-stage carcinogenesis model, we found that HPV16 E2/E4/E5 mice develop tumors rapidly after DMBA-TPA treatment. The significant difference of disease-free survival indicates early development of papillomas and lesions in cK5-rtTA/Tet-E2/E4/E5 mice. Earlier development of papillomas and lesions in cK5-rtTA/Tet-E2/E4/E5 mice as well as development of more papillomas show that E2/E4/E5 induces aberrant proliferation and produces a more aggressive disease. However, this is not a dramatic effect and is a limitation of this particular model.

To further explore the potential pathways and downstream molecules in E2/E4/E5 subtype, we employed ssGSEA to define significant gene sets in E2/E4/E5 subtype, which includes REACTOME signaling by FGFR3 mutants, REACTOME FGFR ligand binding and activation, and REACTOME FGFR4 ligand binding and activation. FGFRs, a class of RTK, dimerize and undergo transphosphorylation of the kinase domain upon ligand binding, leading to the recruitment of adapter proteins and initiating downstream signaling. Because of the ability of FGFR signaling to induce cell proliferation, migration and survival, FGFRs are readily co-opted by cancer cells [43]. FGFR signaling was known to activate the PI3K-AKT signaling pathway by recruitment of GRB2-associated binding protein 1 (GAB1) to the FRS2 complex [44]. In previous study, FGFRs were identified with high mutation incidence in HPV positive HNSCC [45], and HPV16 E5 induced FGFR2c and triggered epithelial-mesenchymal transition (EMT) in cervical cancer [46]. Targeting the FGFR signaling pathway can be a promising therapeutic strategy, but early-phase clinical trials revealed substantial complexity to targeting aberrant FGFR signaling. The PI3K-AKT signaling pathway is frequently implicated in mediating resistance to FGFR inhibitors by activating mTOR and consequently altering cell metabolism and antiapoptotic signals [44]. Thus, in our study, we introduced a mTOR inhibitor and show that addition of pan FGFR inhibitor AZD4547 alone and in combination with mTOR inhibitor rapamycin inhibited cell growth associated with E2/E4/E5 in HPV positive cancers. Besides, we noted broad response to FGFR inhibitor AZD4547 in both E2/E4/E5 expressing cell lines and E6/E7 expressing cell lines (Supplementary Fig. S9). We suspect that there is diversity of activated pathways within these broad subtypes, as well as heterogeneity in gene dosage of HPV genes, such that high E2/E4/E5 expression alone is likely not the sole determinant of FGFR inhibitor response. However, this does not discount the fact that E2/E4/E5 cell lines are responsive, but indicates that there are other phenotypes which may determine responsiveness. The implication of these data is that E2/E4/E5 subtype of HPV related cancers may be targetable using existing targeted therapeutic strategies, providing a potential option for less toxic or de-escalated therapy for patients with these tumor types.

Prior studies attempted to identify subtypes in HNSCC treated HPV as a discrete variable (positive vs. negative). In one study, Zhang et al. [47] identified two subtypes of HPV positive head and neck cancer based on expression patterns from RNAseq data, and found to strongly correlate with viral characteristics, CNAs and oncogenic PIK3CA mutations. Gleber-Netto et al. [48] also revealed relevant variations in HPV function among HPV positive patients, and found that E1^E4 expression was associating with key pathways in HPV positive patients and cell lines and might regulate the tumor radiation treatment response. During our study, we revealed the association between HPV gene expression and integration initially in HPV positive cancer, and validated the biological patterns of a new subtype defined by HPV gene expression in vitro and in vivo. However, there are further questions that lead from these data. Although the changes of cell cycle in E2/E4/E5 cells related to p53 suggests that E2/E4/E5 may contribute to radioresistance depending on the function of p53, it is unclear whether E2/E4/E5 bind to and degrade p53 using the same mechanism of oncoprotein E6 [34]. Future studies will be necessary to determine the precise mechanisms and downstream pathways of E2/E4/E5 and improve our understanding of HPV function and its role in episomal virus tumorigenesis.

In conclusion, we found that concurrent expression of HPV16 E2/E4/E5 genes represents an alternative pathway to HPV-related carcinogenesis and defined an alternative mechanism which may provide an opportunity for a novel understanding of HPV carcinogenesis and may allow for rational therapeutic design to interrupt networks involved in cancer development.

## Materials and methods

### Identification of HPV integration and HPV gene expression from RNAseq data

Both HPV positive HNSCC and CESC datasets from TCGA, as well as an independent primary HPV positive OPSCC dataset from JHH, were used in this study. Methods for sequencing and data processing of RNA using the RNAseq protocol have been previously described for TCGA [4, 6] and JHH [49]. Of note all tumors from the JHH cohort demonstrated p16 immunohistochemical staining, regardless of HPV gene expression. Integration and expression of HPV genes were identified by taking reads in RNAseq data aligned to a combined database of human reference genome and high-risk HPV16, HPV33, HPV35 reference genomes using MapSplice (https://github.com/favorov/viruses-in-sequencing) [50]. Samples were counted in as HPV positive by RNAseq if at least ten reads mapping to the HPV genome were present. Read counts per HPV gene were determined and collapsed to unique reads per HPV gene (E1, E2, E4, E5, E6, E7, L1 and L2) among variants of HPV [6] (Supplementary Tables S1, S4, S5). To analyze patterns of differential HPV gene expression, HPV gene read counts were standardized within each tumor (mean = 0, SD = 1). Then, genes were standardized (mean = 0, SD = 1) [6]. Clustering of these normalized expression values was performed using R/Bioconductor version 3.3.2.

### Cell culture

Colon cancer cell line HCT116 p53+/+ was purchased directly from the American Type Culture Collection (ATCC, Manassas, VA). Colon cancer cell line HCT116 p53−/− was a gift from Vogelstein’s lab. Cervical cancer cell line Caski and SiHa, and HPV positive HNSCC cell lines UM-SCC-047, UPCI-SCC-090, UM-SCC-104, and 93-VU-147T were gifts from Sidransky’s lab. HPV positive HNSCC cell line UD-SCC-2, and HPV negative HNSCC cell line Detroit 562 and CAL27 were gifts from Gutkind’s lab. HCT116 p53+/+, HCT116 p53−/−, SiHa, UM-SCC-047, UPCI-SCC-090, UM-SCC-104, UD-SCC-2, 93-VU-147T, Detroit 562, and CAL27 were grown in DMEM (Sigma-Aldrich, St. Louis, MO). Caski was grown in RPMI-1640 Medium (Sigma-Aldrich). All media were supplemented with 10% FBS (Sigma-Aldrich) and 1% penicillin, streptomycin (Sigma-Aldrich), and cells were cultivated at 37 °C with 5% CO_2_.

Primary oral keratinocytes isolation and culture was performed as described [51]. Primary oral keratinocytes were grown in defined keratinocyte serum free media (SFM, Invitrogen, Carlsbad, CA) supplemented with 1% antibiotics, 5 ng/ml mouse epidermal growth factor (EGF, Invitrogen) and 2 × 10^−11^ M cholera (Sigma-Aldrich) at 37 °C with 5% CO_2_.

### cK5-rtTA transgenic mice

The cK5-rtTA transgenic FVB/N mice have been previously described [52]. cK5-rtTA/Tet-E6/E7 mice were generated by collaborators as previously described [20]. For the generation of cK5-rtTA/Tet-E2/E4/E5 mice, we used a similar method. The open reading frame from the HPV16 E2/E4/E5 coding region was amplified from the HPV16 genome and cloned downstream of the seven Tet-responsive element (Tet-O7) in a modified pBSRV vector. The fragment containing the expression cassette was isolated by PmeI digestion from vector DNA and purified for micro-injection into FVB/N mouse fertilized oocytes. Founders were identified for the presence of the transgene by screening genomic DNA from tail biopsies using a PCR reaction. The presence of the E6/E7 transgenes were determined by PCR with the following primers: forward sequence 5′-TGATCTCTACTGTTATGAGCAATTAAATG-3′, reverse sequence 5′-TGTCCGGTTCTGCCTGTCC-3′. The presence of the E2/E4/E5 transgenes were determined by PCR with the following primers: forward sequence 5′-GATTTAAACATATTAACCACCAAGT-3′, reverse sequence 5′-TATATGTGTCCAGTTTGTATAATGC-3′. The presence of the rtTA transgene was determined by PCR with the following primers: forward sequence 5′-CCGGATCCACCATGCCTAAGAGCCCACG-3′, reverse sequence 5′-ATCTGAATGTACTTTTGCTCCATTGCGAT-3′. A similar number of wild-type mice (cK5-rtTA) as well as transgenic mice (cK5-rtTA/Tet-E2/E4/E5) received doxycycline treatment at the same time. Both male and female mice were used in the studies. Doxycycline was administered after 3 weeks old in the water with a concentration at 1 g/L. The epidermal thickness was compared in sections stained with H&E, tracing parallel lines between basal layer to the cornified layer using at least ten measurements per image in each mouse per group. Two-stage chemical induced carcinogenesis was performed essentially as previously described [53]. Briefly, mice were shaved in the back and tumors were initiated by the topical treatment with a single dose of DMBA (100 μg/200 μl in acetone, Sigma-Aldrich) followed by the tumor promotion phase in which mice were treated twice weekly with TPA (12.5 μg/200 μl in acetone, Sigma-Aldrich) for 24 weeks. The lesion numbers in each mouse were measured twice weekly. Animals were euthanized after the last TPA treatment, and treated skin areas were fixed in Z-fix (Anatech Ltd, Battle Creek, MI) overnight and then transferred to 70% ethanol. Fixed tissues were embedded in paraffin, sectioned to a thickness of 4 μm and stained with H&E. Histology slides were scanned with Scanscope digital microscope (Aperio, Vista, CA).

### Statistics

Statistical analyses were performed using R/Bioconductor version 3.3.2. The Fisher’s exact test was used to explore the association between mutations and CNAs, and two subtypes, and the association between number of mice with papillomas and two groups. The Log-rank test was applied to explore the association of groups and disease-free survivals. The two-sided Student *t* test was performed to analyze normally distributed data. *P* < 0.05 was treated as statistical significance. Chou Talalay combination index were calculated using CompuSyn software to examine synergistic effects (combination index score < 0.5).

## Supplementary information

Supplementary Data

Figure S1

Figure S2

Figure S3

Figure S4

Figure S5

Figure S6

Figure S7

Figure S8

Figure S9

Table S1

Table S2

Table S3

Table S4

Table S5
