# Supplementary material for: HPV E2, E4, E5 drive alternative carcinogenic pathways in HPV positive cancers
Source: Oncogene. 2020 Aug 26;39(40):6327–39. doi: 10.1038/s41388-020-01431-8 (PMC7529583; doi:10.1038/s41388-020-01431-8)
Supplement: Supplementary file 1 — Supplementary Data [file 41388_2020_1431_MOESM1_ESM.docx]

***Supplementary Information***

**HPV E2, E4, E5 drive alternative carcinogenic pathways in HPV positive cancers**

Shuling Ren, Daria A. Gaykalova, Theresa Guo, Alexander V. Favorov, Elana J. Fertig, Pablo Tamayo, Juan Luis Callejas-Valera, Mike Allevato, Mara Gilardi, Jessica Santos, Takahito Fukusumi, Akihiro Sakai, Mizuo Ando, Sayed Sadat, Chao Liu, Guorong Xu, Kathleen M. Fisch, Zhiyong Wang, Alfredo A. Molinolo, J. Silvio Gutkind, Trey Ideker, Wayne M. Koch, Joseph A. Califano

**Supplementary Methods**

***mRNA expression analysis***

Methods for RNA sequencing have been previously described [1, 2]. The analysis of mRNA expression datasets was performed using R/Bioconductor version 3.3.2. Standard statistical tests, such as the Student t test are not suited for high-throughput studies, but outlier analysis based on robust centering and scaling of the data may be used to find differential expressed genes [3]. A modified outlier approach was used, in which minimum change levels were set for the definition of an outlier [4, 5]. Expression data from normal samples were used to establish the baseline for each gene. Outlier p value were calculated for right-tail cases, which allowed the definition of outliers that were overexpressed on tumors [6]. We carried out unsupervised hierarchical clustering on HPV positive HNSCC RNAseq data from TCGA and HPV positive OPSCC RNAseq data from JHH separately using all statistically significant outlier genes in E2/E4/E5 activated, non-integrated samples and E6/E7 activated, integrated samples compared with normal samples. Additionally, in HPV positive HNSCC TCGA dataset, we conducted supervised clustering using the outlier genes mentioned above looking for differential patterns of mRNA expression between E2/E4/E5 subtype and E6/E7 subtype.

***Identification of mutations and CNAs***

To characterize mutations and CNAs in different HPV genes subtypes, we applied cBioPortal (http://www.cbioportal.org). 41 significantly mutated genes based on q value identified using the MutSigCV algorithim in TCGA HNSCC dataset were chosen. Frequencies of mutations and CNAs were shown in each subtype and p values were calculated to explore the association between mutations and CNAs, and two subtypes. Similarly, 15 significantly mutated genes were selected in TCGA CESC dataset. Frequencies and p values were also shown by side.

***Survival analysis***

We performed a disease-free survival analysis in TCGA HPV positive HNSCC cohort. Clinical data were downloaded from TCGA website (https://portal.gdc.cancer.gov). Recurrence time was the time in months from tumor biopsy to recurrence, death or loss to follow-up. The follow-up time is 0-105.81 months. HPV subtypes were defined depending on results from HPV genes read counts (Fig. 1A). 37 tumors with higher expression of E2/E4/E5 were classified into E2/E4/E5 subtype, while 28 tumors with higher expression of E6/E7 were included in E6/E7 subtype. A disease-free survival analysis was also performed in cK5-rtTA transgenic mice. The time in weeks from the start of treatment to the appearance of lesions or death was recorded. The follow-up time is 18-24 weeks. 10 mice in cK5-rtTA group and 8 mice in cK5-rtTA/Tet-E2/E4/E5 group were included.

***RT-qPCR***

Total RNA was isolated from tumor tissues and cells using RNeasy Plus Mini Kit (Qiagen, Valencia, CA). cDNA was obtained using High-Capacity cDNA Reverse Transcription Kit (Applied Biosystems, Waltham, MA). RT-qPCR primers and probes for each HPV genes (E2, E4, E5, E6, E7) were designed using PrimerQuest Tool (https://www.idtdna.com/Primerquest/Home/Index) from Integrated DNA Technologies (IDT, Coralville, IA) and validated using regular PCR. Optimized primers and probe set were purchased from IDT and sequences of each set are provided in Supplementary Table S2. RT-qPCR amplification was performed using Platinum Taq DNA Polymerase (Invitrogen, Carlsbad, CA). Absolute quantitation method with standard curve was used to measure the exact copy number of each HPV gene in JHH dataset (Fig. 1d). Relative quantification method with comparative CT value was used in cell lines experiments to validate the transfection efficiency (Fig. 2a-d) and relative HPV16 gene expression in HPV positive cancer cell lines (Fig. 6b). Relative expression levels were normalized to an endogenous control 18S using Hs99999901_s1 18S TaqMan Gene Expression Assays (Applied Biosystems, Waltham, MA). Error bars indicate SEM of 3 independent experiments.

***IHC and IF***

Staining were performed on 5μm sections that were melted for 4 hours at 65°C. All sections were deparaffinized and gradually hydrated from 100% to 70 % ethanol. After washing the slides in abundant distilled water, the tissues were submitted to heat-induced epitope retrieval using IHC Antigen Retrieval Solution (Invitrogen, Carlsbad, CA). Endogenous peroxidase was blocked with 3.5% H_2_O_2_. Protein blocking was performed using 1% BSA in PBS (Sigma-Aldrich, St. Louis, MO) and the cells were treated with 0.015% Triton (Sigma-Aldrich, St. Louis, MO) for 20 minutes at room temperature.

For IHC, the sections were incubated overnight with Ki67 (#ab15580, 1:500, Abcam, Cambridge, UK) at 4°C and 30 minutes with anti-rabbit biotinylated secondary antibody (#BA-1400, 1:400, Vector Laboratories, Burlingame, CA) at room temperature. ABC Kit (Vector Laboratories, Burlingame, CA) was used as detection system and 3,3’-diaminobenzidine (Vector Laboratories, Burlingame, CA) was used to reveal antigen-antibody reactions. Mayer’s hematoxylin was used for counterstain.

For IF, the sections were incubated overnight with the Ki67 (#ab15580, 1:500, Abcam, Cambridge, UK) and cytokeratin 1 (#MA1-35367, 1:2 000, Invitrogen, Carlsbad, CA) at 4°C followed by 60 minutes incubation with Alexa Fluor 488-labeled anti-mouse IgG, Alexa Fluor 647-labeled anti-rabbit IgG and DAPI (#A11029, #A21244 and #D3571, Invitrogen, Carlsbad, CA). Ki67 was linked to Alexa Fluor 647, signaling in red, and cytokeratin 1 (green) and DAPI (blue) was applied to contrast the tissues.

All sections were gradually dehydrated from 70% to 100% ethanol and mounted with mounting media.

***Western blot***

Cells were collected in RIPA lysis buffer (50 mM Tris–HCl pH 7.4, 0.5% sodium deoxycholate, 1% Nonidet P-40, 0.1% SDS, 150 mM NaCl, 2 mM EDTA, 50mM NaF) supplemented with protease inhibitor (Roche, Basel, Switzerland) and phosphatase inhibitor (Roche, Basel, Switzerland). Protein quantification was performed by using the BCA Protein Assay Kit (Bio-Rad, Hercules, CA). Equal amounts of protein were loaded onto Mini-PROTE-AN TGX gels (Bio-Rad, Hercules, CA), transferred to polyvinylidene fluoride membranes (Millipore, Burlington, MA) and blotted using specific antibodies. Antibody detection was achieved by ECL detection reagent (Thermo Scientific, Carlsbad, CA). Antibodies against pFGFR1 (#2544, 1:1 000), FGFR1 (#9740, 1:2 000), pFRS2 (#3861, 1:1 000), pAKT^T308^ (#2965, 1:1 000), AKT (#9272, 1:1 000), pS6 (#4858, 1:5 000) and S6 (#2317, 1:2 000) were purchased from Cell Signaling Technologies (Danvers, MA). Antibody against pFGFR3 (#ab155960, 1:1 000) was purchased from Abcam (Cambridge, UK). Antibody against FGFR3 (#sc-13121, 1:500) was purchased from Santa Cruz (Dallas, TX). Antibody against FRS2 (#GTX103288, 1:500) was purchased from GeneTex (Irvine, CA). Anti-GAPDH (#2118, 1:10 000, Cell Signaling Technology, Danvers, MA) was used as the loading control. HRP-conjugated goat anti-mouse (#1010-05, 1:20 000, SouthernBiotech, Birmingham, AL) or anti-rabbit antibodies (#4010-05, 1:20 000, SouthernBiotech, Birmingham, AL) were used as secondary antibodies. Results show a representative experiment of three times.

***Plasmids, viruses and siRNAs***

pCEFL2-E2/E4/E5 was made by cloning E2/E4/E5 into pCEFL2 by collaborators in Gutkind’s Lab and pCEFL2-E6/E7 was produced by recombination pENTR-E6/E7 (Gutkind’s Lab) with pCEFL2. Transient transfection was performed using the plasmid of interest, X-tremeGENE 9 DNA transfection reagent (Roche, Basel, Switzerland) and Opti-MEM reduced serum medium (Gibco, Grand Island, NY). pLenti-E2/E4/E5 was made by recombination pCEFL2-E2/E4/E5, pENTR and pLenti sequentially, and pLenti-E6/E7 was made by recombination pENTR-E6/E7 with pLenti. Lentiviruses were produced in 293T cells by co-transfecting pLenti-E2/E4/E5 or pLenti-E6/E7 with packaging vectors and enveloping vectors using Turbofect (Thermo Scientific, Carlsbad, CA) according to the manufacturer’s instructions. Viral supernatants were collected 72 hours after transfection and concentrated using 20% sucrose in TNE buffer (Tris pH7.5 10 mM, NaCl 150 mM, EDTA 5 mM). Virus-containing pellets were gently resuspended in TNE buffer and added dropwise on cells in the presence of media with 8 μg/mL polybrene. Lentivirally infected cells were selected with 1 μg/mL puromycin at 72 hours following infection. siRNAs targeting each HPV gene (E2, E4, E6, E7) were designed using siDESIGN Center (http://dharmacon.gelifesciences.com/design-center/) from Dharmacon (Lafayette, CO). Downregulation was performed using siRNA of interest, Lipofectamine RNAiMAX transfection reagent (Thermo Scientific, Carlsbad, CA) and Opti-MEM reduced serum medium. RNA collection was employed 48 hours after transient transfection. Upregulation or downregulation efficiency was determined by RT-qPCR.

***Cell viability assays***

Cells were seeded in five times repeat wells in 96-well plates and grown under log phase growth conditions. For proliferation assays, Caski cells were transfected with the corresponding plasmid and siRNA, and HCT116 p53+/+ and HCT116 p53-/- cells were infected with the corresponding lentivirus upregulating E2/E4/E5 and stable expression cell lines were established, then cell numbers were measured every 24 hours. For AZD4547 viability assays, UD-SCC-2, UPCI-SCC-090, UM-SCC-104 and Caski cells were treated with DMSO (untreated group), FGFR inhibitor AZD4547 (Selleckchem, Houston, TX) or/and mTOR inhibitor rapamycin (LC Labs, Woburn, MA) for 3 days. Cell viability was measured using the Vita-blue cell viability reagent (Biotool, Houston, TX) as described by the manufacturer. Briefly, 10 μl Vita-Blue cell viability reagent per 100 ul culture medium were added to each well and incubated at 37°C for 1 hour. Endpoint fluorescence (Ex=540 nm, Em=580 nm) was measured on Synergy HTX multi-mode reader (BioTek, Winooski, VT). Cell viability was normalized to fluorescence of the first day. The data displayed are representative of at least three experiments performed in five times repeat.

***Colony formation assays***

Colony formation assays were performed as previously described [7]. Primary oral keratinocytes from cK5-rtTA mice and cK5-rtTA/Tet-E2/E4/E5 mice were seeded into 6-well plates (1 000 cells per well) and incubated with doxycycline (Sigma-Aldrich, St. Louis, MO) for 2 weeks. Then the colonies were fixed with 4% paraformaldehyde and stained with crystal violet. Each experiment was repeated independently in triplicate.

***Cell cycle assays***

FACS was used to analyze cell cycle changes 24 hours following irradiation as previously described [8]. Propidium iodide (PI, Roche, Basel, Switzerland) was used as a marker for DNA content. Freshly trypsinized cells were fixed in ice cold 70% ethanol and kept at 4°C overnight. Before analysis cells were washed in ice cold PBS and resuspended in PBS containing 20 μg/ml PI, 0.1% Triton X-100 (Sigma-Aldrich, St. Louis, MO), and 200 μg/ml RNase A (Sigma-Aldrich, St. Louis, MO). Labeled cells were analyzed using a FACSCalibur machine (BD Biosciences, San Jose, CA). Percentages of cells in each phase of the cell cycle were quantified using the FlowJo software. At least three times of independent experiments were performed.

***Apoptosis assays***

FACS was used to analyze apoptosis changes 48 hours following irradiation. Annexin V-FITC Detection Kit (Biotool, Houston, TX) was used. Annexin V-FITC labels phosphatidylserine sites on the membrane surface while PI stains necrotic cells. Freshly trypsinized cells were washed in ice cold PBS, suspended in binding buffer and stained with Annexin V-FITC and PI. Labeled cells were analyzed using a FACSCalibur machine (BD Biosciences, San Jose, CA). Percentages of cells in right upper quadrant and right bottom quadrant were quantified using the FlowJo software. At least three times of independent experiments were performed.

***ssGSEA***

Gene set enrichment analysis (GSEA) is the method to determine whether one set of genes are randomly distributed throughout another ranked list of genes or primarily found at the top or bottom. Candidate genes are ranked by their differential expression between two phenotypes [9]. Here we applied an extended version of conventional GSEA in order to produce an enrichment score in a single sample as described previously [10, 11]. Such a score is necessary if one is to make a predictive call on a single sample without reference to a larger group of samples. In this approach, the genes are ordered based on either absolute expression or the relative changes with respect to the baseline level. We performed ssGSEA in TCGA HPV positive HNSCC dataset and JHH HPV positive OPSCC dataset respectively to define differentially expressed gene sets that are unique to E2/E4/E5 expressing tumors as compared to E6/E7 expressing tumors. We chose overlapped gene sets significantly expressed in each subset and used Morpheus for visualization and making heatmaps (https://software.broadinstitute.org/morpheus/).

***Study approval***

All JHH tissue samples were collected from the Johns Hopkins Tissue Core under an approved Institutional Review Board protocol (#NA_00036235) and written informed consent was received from participants prior to inclusion in the study. All mice experiments were conducted in accordance with NIH guidelines for the use of live animals under an approved Institutional Animal Care and Use Committee protocol (#S16200) of University of California San Diego.

**Supplementary Figures**

**Figure S1 Unsupervised clustering of HPV positive samples in TCGA HNSCC dataset (a) and JHH OPSCC dataset (b) using all statistically significant outlier genes in E2/E4/E5 activated, non-integrated samples and E6/E7 activated, integrated samples compared with normal samples.** Annotations were made with HPV integration status and HPV gene expression subtypes.

**Figure S2 Supervised clustering of mRNA expression of significant outlier genes (in rows) in all HPV positive samples (in columns) in TCGA HNSCC dataset.** The columns of the heatmap were supervised by HPV gene expression subtypes.

**Figure S3 Significant alternated events in E2/E4/E5 and E6/E7 subtypes. Alteration events for significantly mutated genes are displayed by samples.** (a) Mutations and CNAs in TCGA HPV positive HNSCC. (b) Mutations and CNAs in TCGA CESC. Two-sided Fisher’s exact test p values accessing the association between each genomic alteration and two subtypes were listed on the right.

**Figure S4 Association between HPV genes subtypes with disease-free survivals in TCGA HPV positive HNSCC cohort.** A disease-free survival analysis was performed using R/Bioconductor version 3.3.2. Recurrence time was defined as the time in months from tumor biopsy to recurrence, death or loss to follow-up. The Log-rank test was applied to explore the association of HPV subtypes and disease-free survivals. E2/E4/E5 group has shorter median survival compared with E6/E7 group (60.94 months vs. 71.22 months), but there was no statistical association between HPV gene expression and prognosis in HNSCC patients (P=0.41).

**Figure S5 Effect of E2/E4/E5 on growth of HNSCC cell lines.** We upregulated E2/E4/E5 expression in two types of HPV negative HNSCC cell lines (Detroit 562, CAL27), and we downregulated E4 expression in two types of HPV positive HNSCC cell lines (UM-SCC-047, UM-SCC-104). We can find that all transfection was successful according to the RT-qPCR results on the right (P<0.05). However, there is no significant change of growth after expressing E2/E4/E5 in HPV negative HNSCC cell lines. And in one out of two HPV positive HNSCC cell lines, we can find the cell growth was significantly decreased after inhibiting E2/E4/E5 expression (P<0.05).

**Figure S6 No significant effects of E2/E4/E5 on apoptosis of HCT116 p53+/+ and HCT116 p53-/- cells were shown.** HCT116 p53+/+ E2/E4/E5 stable expression cell line and HCT116 p53-/- E2/E4/E5 stable expression cell line were established. Apoptosis was detected by FACS with PI and Annexin V-FITC staining. (a, b) On the left, representative apoptosis diagrams of control cells and irradiated cells 48 hours after 6 Gy irradiation. On the right, the percentages of apoptosis cells analyzed using FlowJo. Data represent mean ± SEM for 3 independent experiments. P values were calculated using two-sided Student t test. ns: no significance.

**Figure S7 cK5-rtTA/Tet-E2/E4/E5 mice developed more papillomas and proliferative lesions than cK5-rtTA mice.** (a) The frequency of mice with papillomas in cK5-rtTA/Tet-E2/E4/E5 group was higher than those in cK5-rtTA group but no significance. P values were calculated using Fisher’s exact test. (b) The number of papillomas in cK5-rtTA/Tet-E2/E4/E5 group was higher than those in cK5-rtTA group. Data represent mean ± SEM. P values were calculated using two-sided Student t test. ns: no significance. (c) The number of lesions in cK5-rtTA/Tet-E2/E4/E5 group was higher than those in cK5-rtTA group. Data represent mean ± SEM. P values were calculated using two-sided Student t test. ns: no significance.

**Figure S8 Significant synergistic effects were found in all four cell lines.** Chou Talalay combination index were calculated using CompuSyn software for each cell line. Combination index score < 0.5 was treated as significant synergistic effect.

**Figure S9 HPV positive HNSCC cell lines showed broad responses to FGFR inhibitor AZD4547.** Tumor growth in both E2/E4/E5 expressing cell lines (UD-SCC-2, UPCI-SCC-090, UM-SCC-104, Caski) and E6/E7 expressing cell lines (UM-SCC-047, 93-VU-147T, SiHa) were inhibited by AZD4547. Growth inhibition was measured at 3 days after treated with AZD4547 (1 uM) and normalized by the non-treated group. Data represent mean ± SEM.

**Supplementary Tables**

**Table S1 Read counts of E1, E2, E4, E5, E6, E7, L1, L2 of HPV16, HPV33, HPV35 in HPV positive samples in TCGA HNSCC dataset.**

**Table S2 Sequences of primers and probe sets of HPV16 genes.**

**Table S3 The list of 42 overlapped gene sets enriched in E2/E4/E5 subtype and 41 gene sets enriched in E6/E7 subtype.**

**Table S4 Read counts of E1, E2, E4, E5, E6, E7, L1, L2 of HPV16, HPV33, HPV35 in HPV positive samples in TCGA CESC dataset.**

**Table S5 Read counts of E1, E2, E4, E5, E6, E7, L1, L2 of HPV16, HPV33, HPV35 in HPV positive samples in JHH OPSCC dataset.**

**References**

1 Cancer Genome Atlas N. Comprehensive genomic characterization of head and neck squamous cell carcinomas. *Nature* 2015; 517: 576-582.

2 Guo T, Gaykalova DA, Considine M, Wheelan S, Pallavajjala A, Bishop JA *et al*. Characterization of functionally active gene fusions in human papillomavirus related oropharyngeal squamous cell carcinoma. *International journal of cancer Journal international du cancer* 2016; 139: 373-382.

3 MacDonald JW, Ghosh D. COPA--cancer outlier profile analysis. *Bioinformatics* 2006; 22: 2950-2951.

4 Ghosh D. Discrete nonparametric algorithms for outlier detection with genomic data. *J Biopharm Stat* 2010; 20: 193-208.

5 Ghosh D. Genomic outlier detection in high-throughput data analysis. *Methods Mol Biol* 2013; 972: 141-153.

6 Gaykalova DA, Vatapalli R, Wei Y, Tsai HL, Wang H, Zhang C *et al*. Outlier Analysis Defines Zinc Finger Gene Family DNA Methylation in Tumors and Saliva of Head and Neck Cancer Patients. *PloS one* 2015; 10: e0142148.

7 Liu C, Guo T, Xu G, Sakai A, Ren S, Fukusumi T *et al*. Characterization of Alternative Splicing Events in HPV-Negative Head and Neck Squamous Cell Carcinoma Identifies an Oncogenic DOCK5 Variant. *Clin Cancer Res* 2018; 24: 5123-5132.

8 Lindgren T, Stigbrand T, Raberg A, Riklund K, Johansson L, Eriksson D. Genome wide expression analysis of radiation-induced DNA damage responses in isogenic HCT116 p53+/+ and HCT116 p53-/- colorectal carcinoma cell lines. *Int J Radiat Biol* 2015; 91: 99-111.

9 Subramanian A, Tamayo P, Mootha VK, Mukherjee S, Ebert BL, Gillette MA *et al*. Gene set enrichment analysis: a knowledge-based approach for interpreting genome-wide expression profiles. *Proceedings of the National Academy of Sciences of the United States of America* 2005; 102: 15545-15550.

10 Barbie DA, Tamayo P, Boehm JS, Kim SY, Moody SE, Dunn IF *et al*. Systematic RNA interference reveals that oncogenic KRAS-driven cancers require TBK1. *Nature* 2009; 462: 108-112.

11 Liberzon A, Birger C, Thorvaldsdottir H, Ghandi M, Mesirov JP, Tamayo P. The Molecular Signatures Database (MSigDB) hallmark gene set collection. *Cell Syst* 2015; 1: 417-425.
