# Supplementary figures and images for: HPV E2, E4, E5 drive alternative carcinogenic pathways in HPV positive cancers
Source: Oncogene. 2020 Aug 26;39(40):6327–39. doi: 10.1038/s41388-020-01431-8 (PMC7529583; doi:10.1038/s41388-020-01431-8)

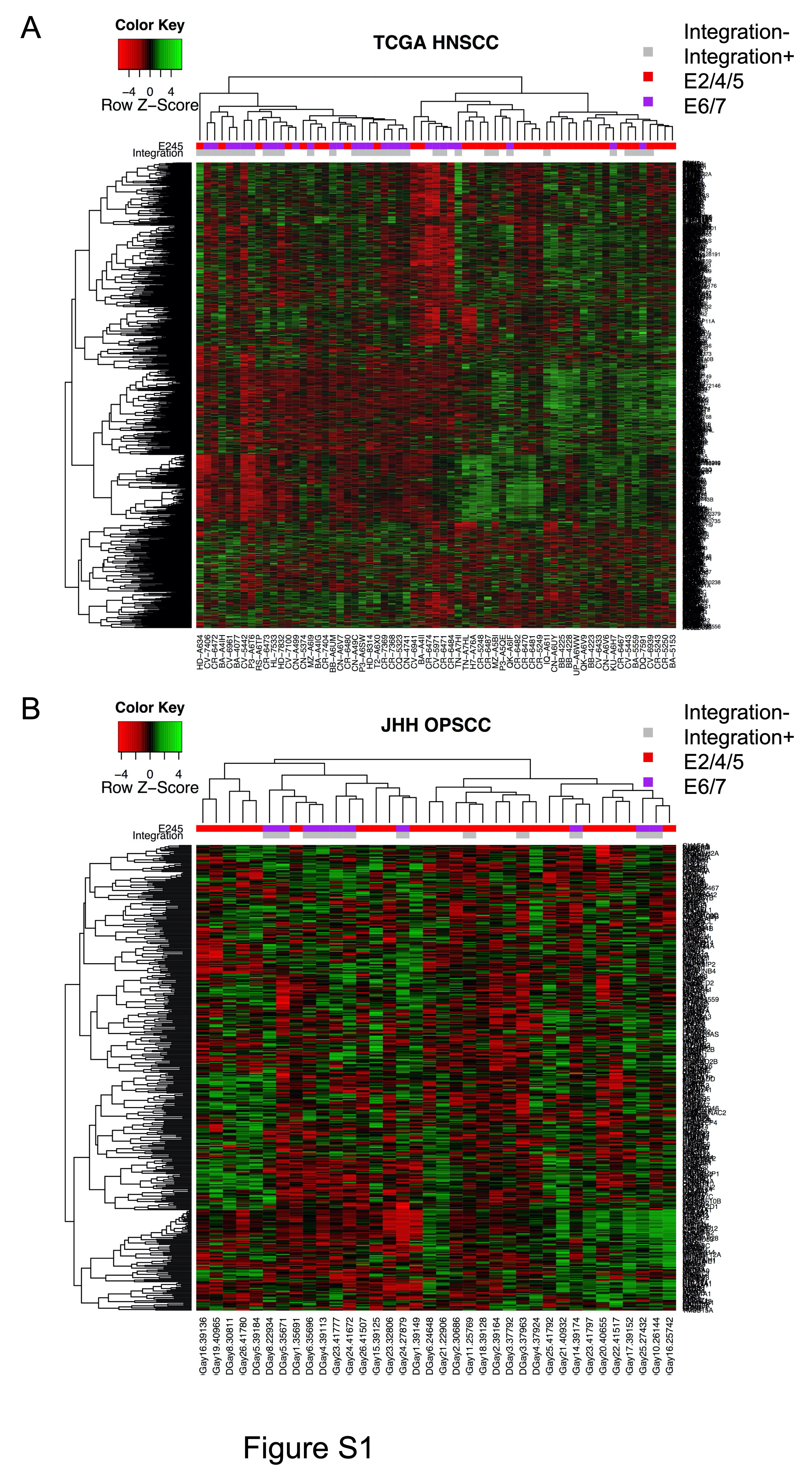

Supplement: Supplementary file 2 — Figure S1 [file 41388_2020_1431_MOESM2_ESM.jpg]

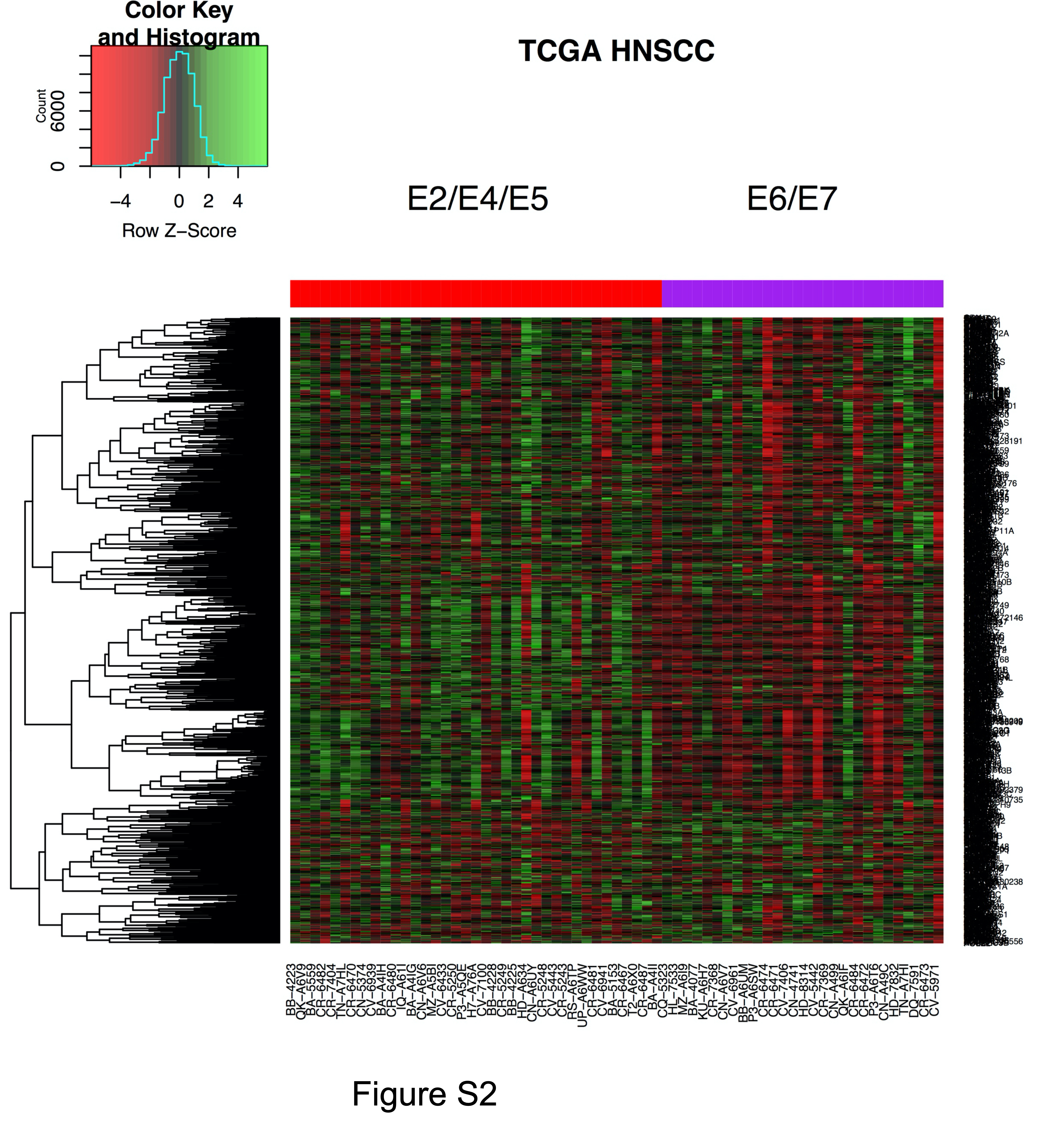

Supplement: Supplementary file 3 — Figure S2 [file 41388_2020_1431_MOESM3_ESM.jpg]

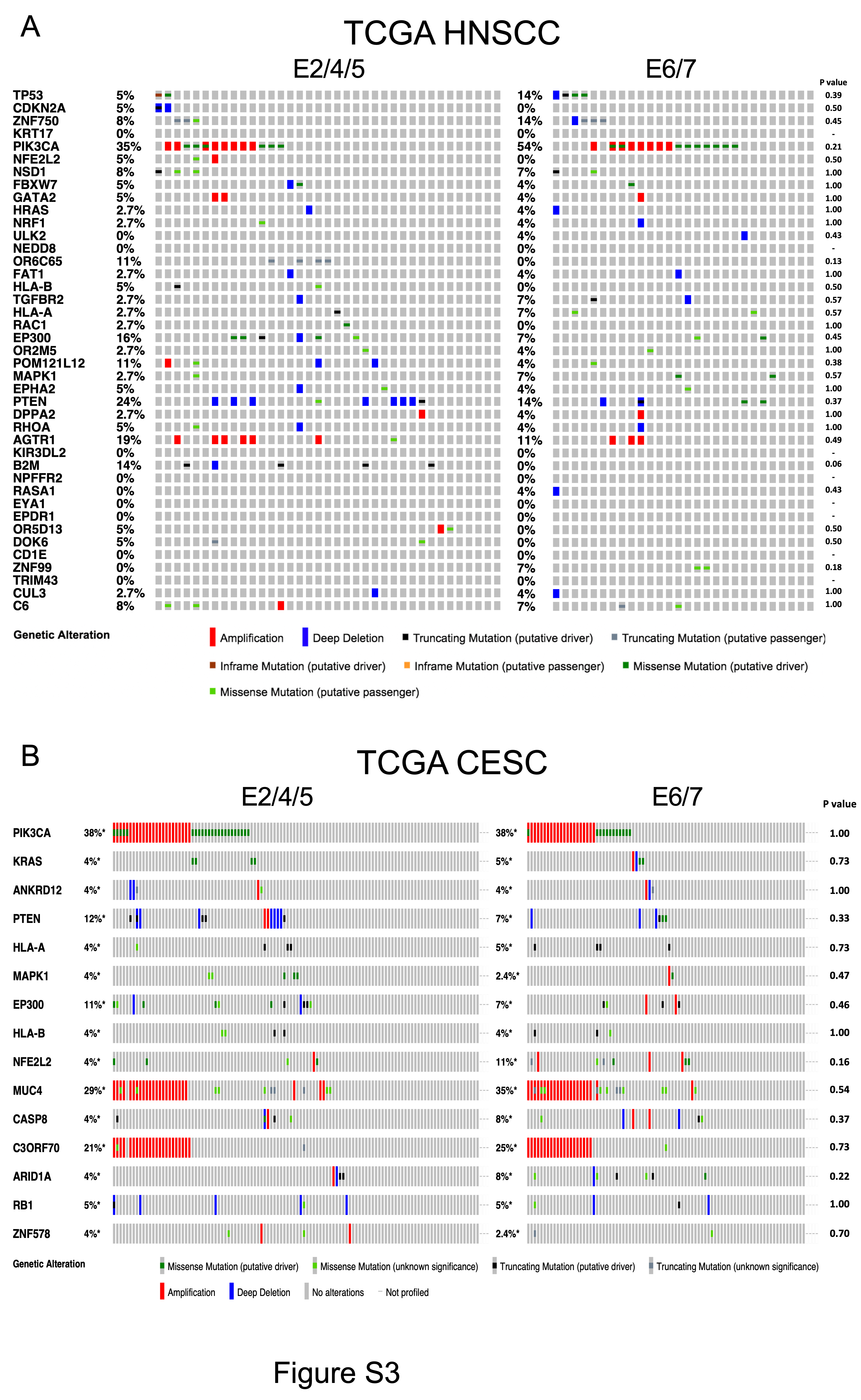

Supplement: Supplementary file 4 — Figure S3 [file 41388_2020_1431_MOESM4_ESM.jpg]

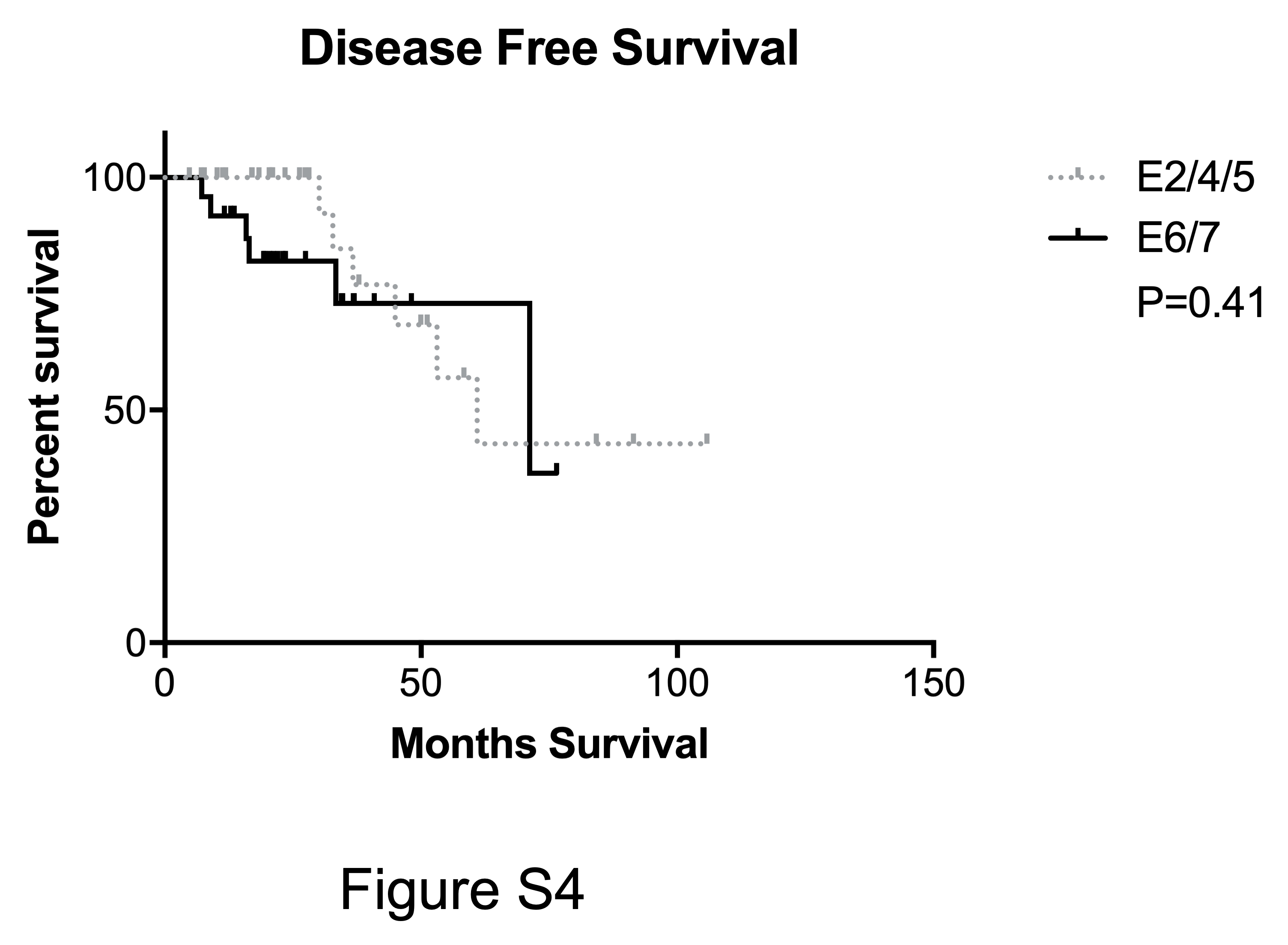

Supplement: Supplementary file 5 — Figure S4 [file 41388_2020_1431_MOESM5_ESM.jpg]

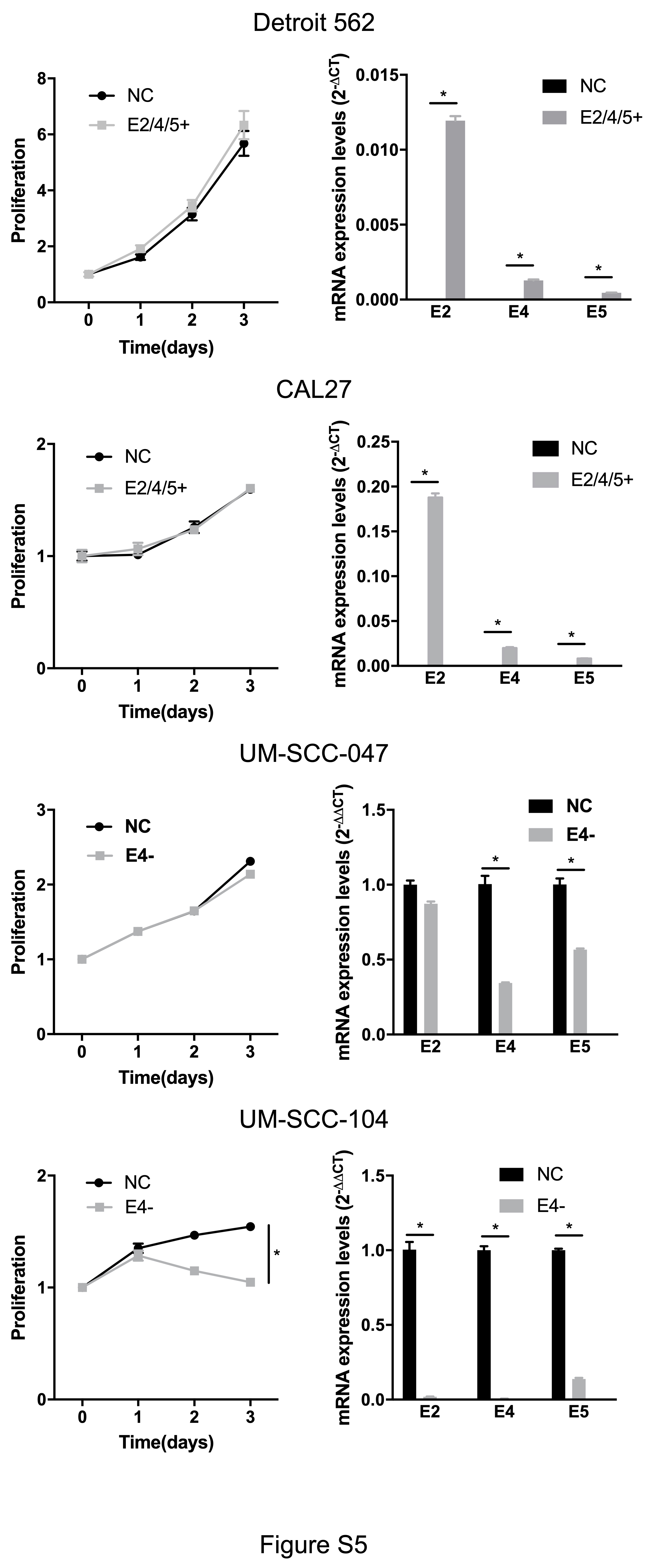

Supplement: Supplementary file 6 — Figure S5 [file 41388_2020_1431_MOESM6_ESM.jpg]

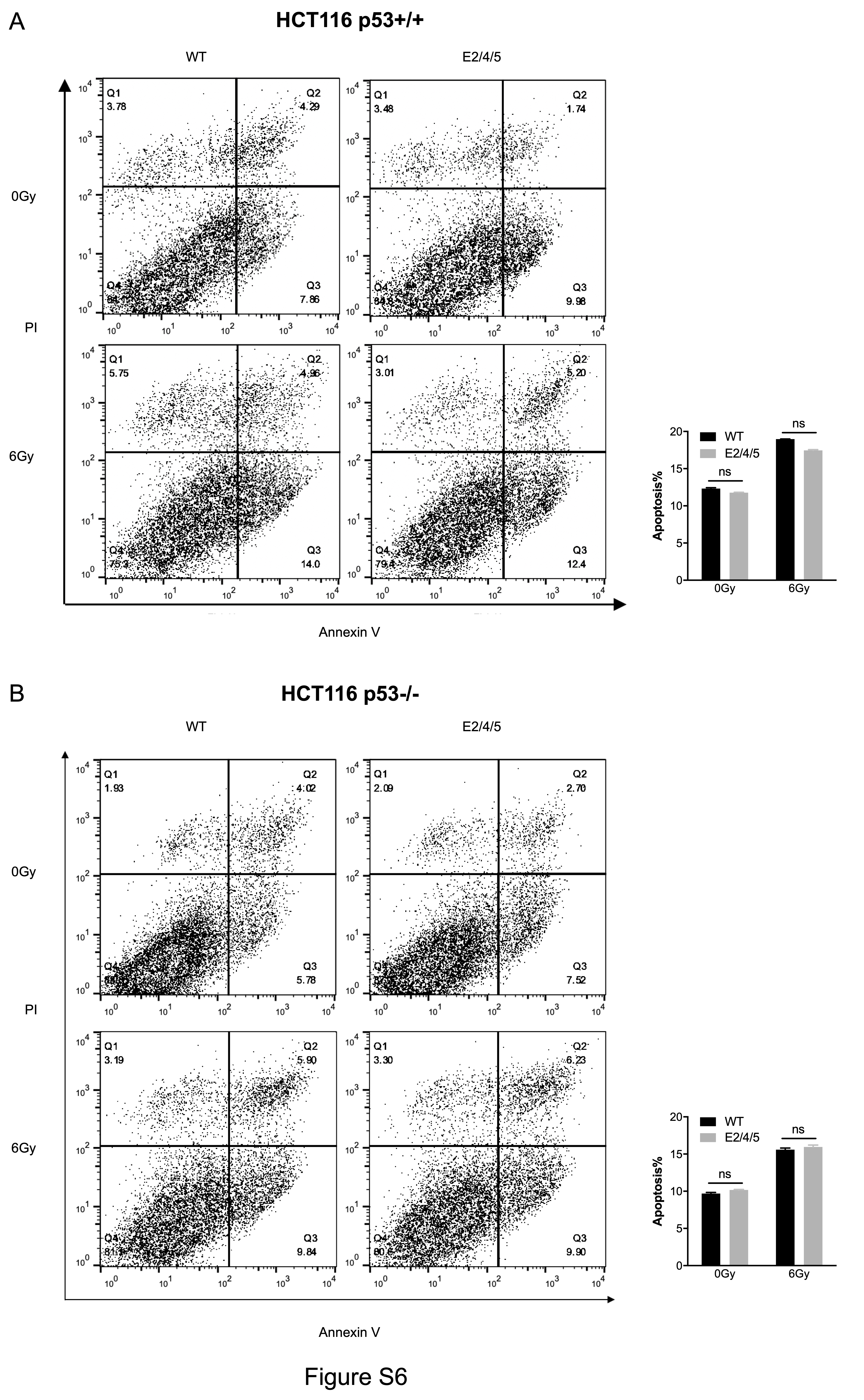

Supplement: Supplementary file 7 — Figure S6 [file 41388_2020_1431_MOESM7_ESM.jpg]

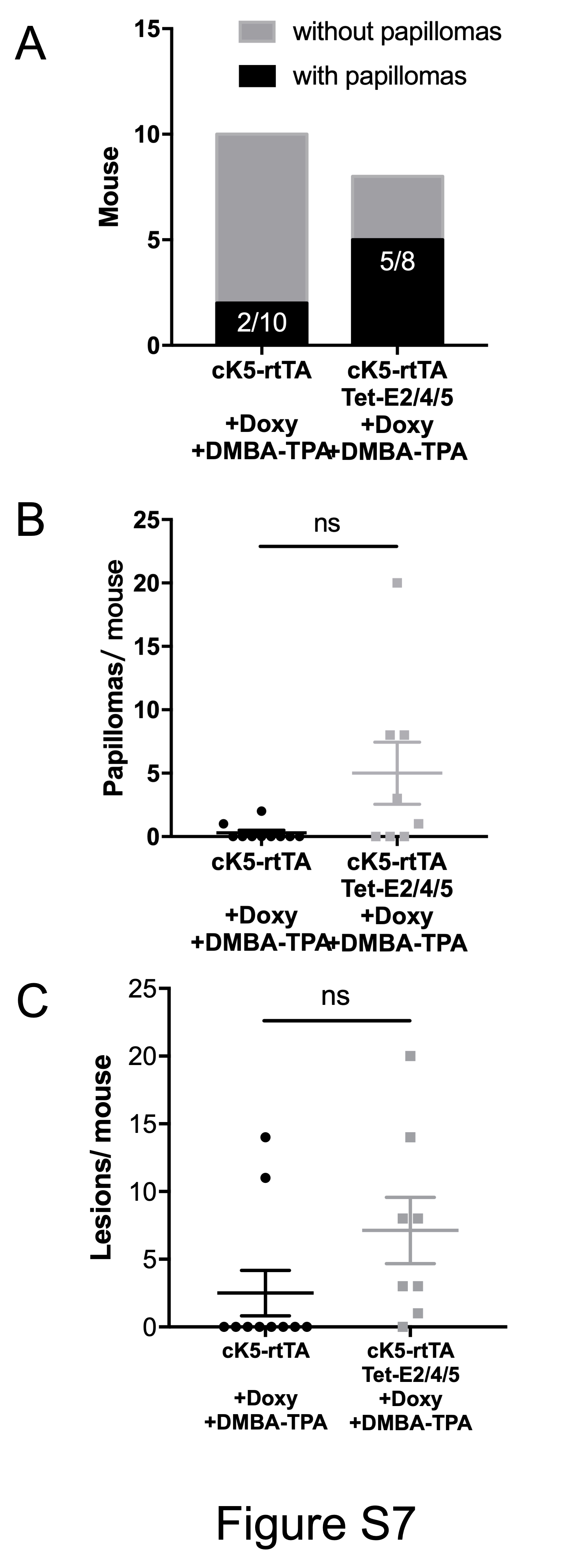

Supplement: Supplementary file 8 — Figure S7 [file 41388_2020_1431_MOESM8_ESM.jpg]

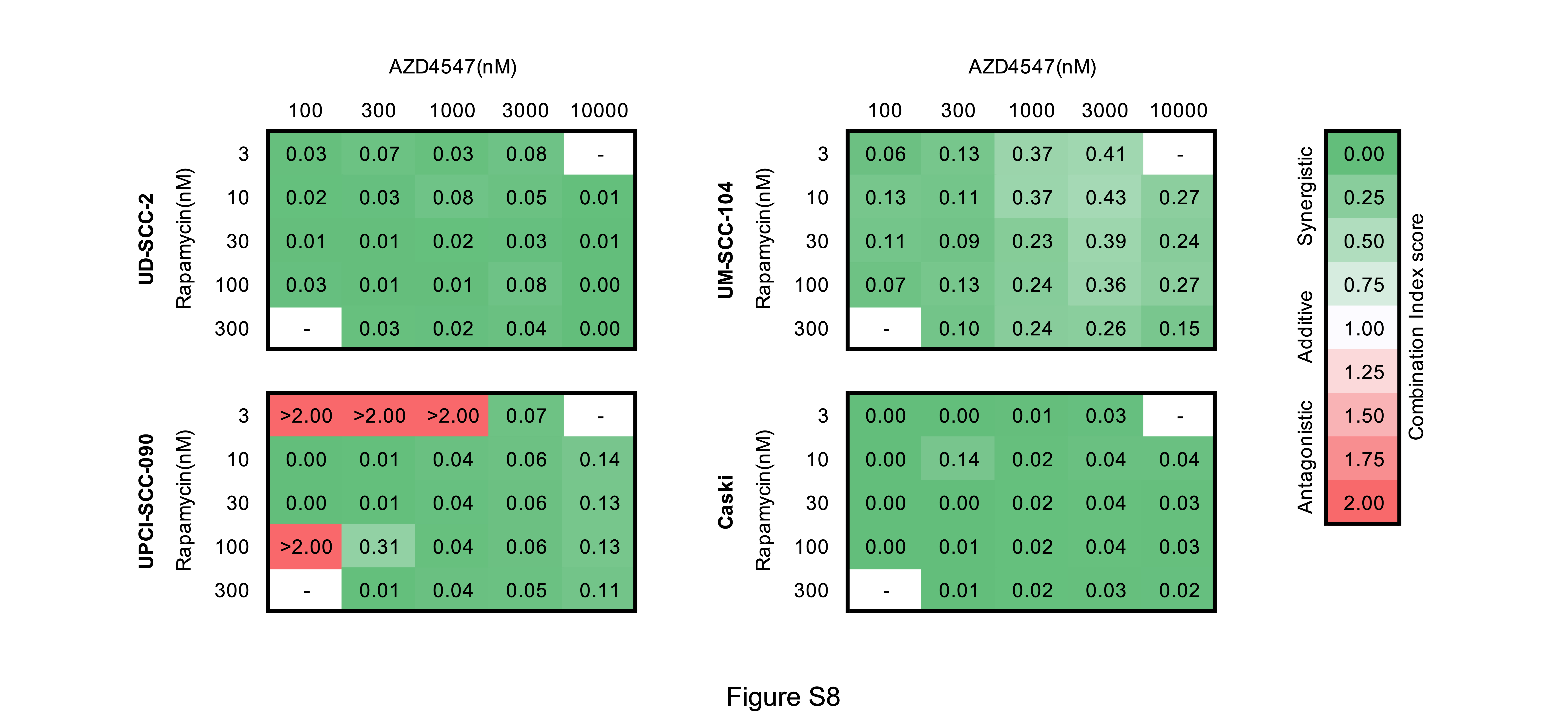

Supplement: Supplementary file 9 — Figure S8 [file 41388_2020_1431_MOESM9_ESM.jpg]

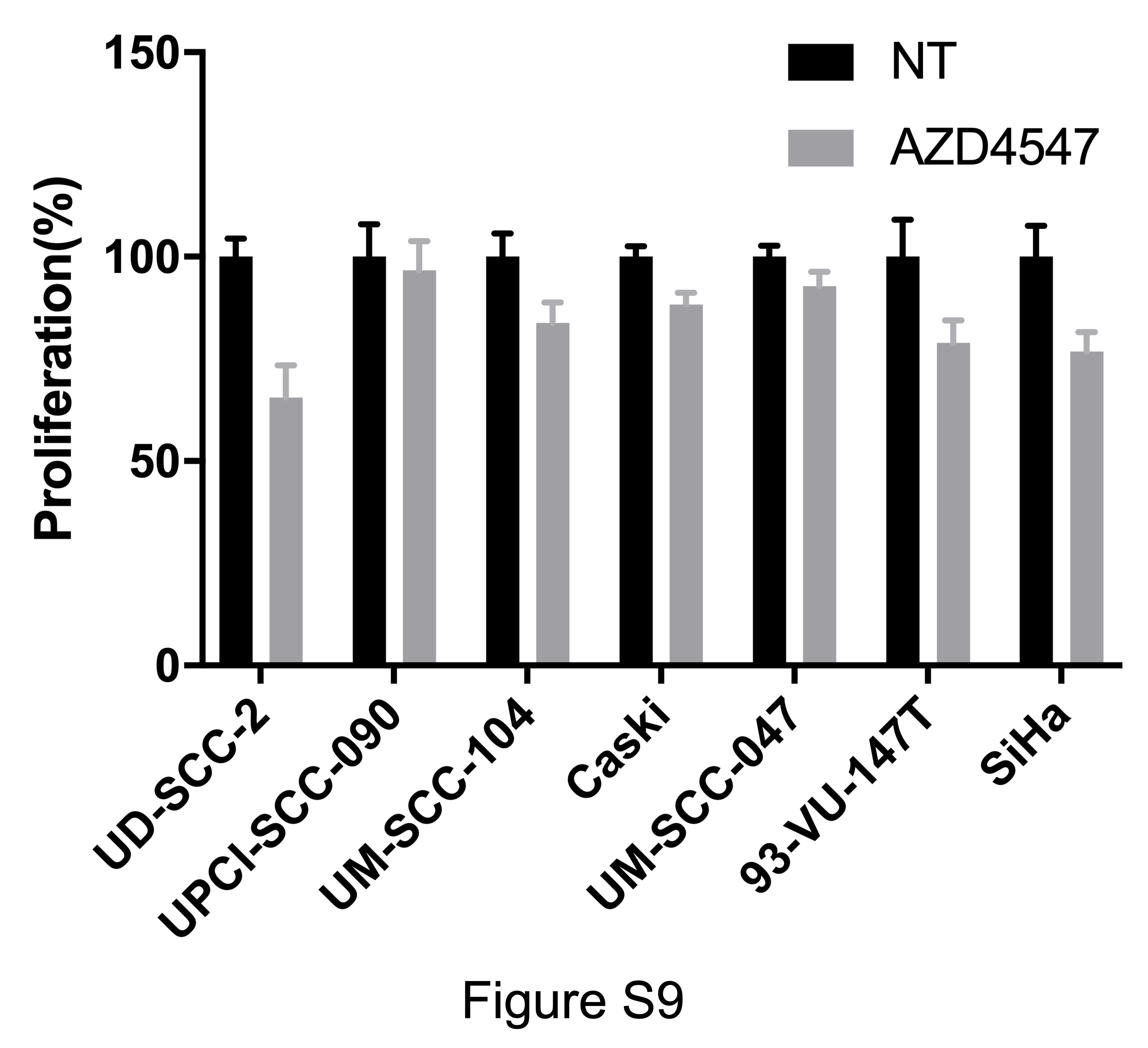

Supplement: Supplementary file 10 — Figure S9 [file 41388_2020_1431_MOESM10_ESM.jpg]
